# A clustered set of three Sp-family genes is ancestral in the Metazoa: evidence from sequence analysis, protein domain structure, developmental expression patterns and chromosomal location

**DOI:** 10.1186/1471-2148-10-88

**Published:** 2010-03-30

**Authors:** Nina D Schaeper, Nikola-Michael Prpic, Ernst A Wimmer

**Affiliations:** 1 Georg-August-Universität, Johann-Friedrich-Blumenbach-Institut für Zoologie und Anthropologie, Abteilung Entwicklungsbiologie, GZMB, Ernst-Caspari-Haus, Justus-von-Liebig-Weg 11, 37077 Göttingen, Germany

## Abstract

**Background:**

The Sp-family of transcription factors are evolutionarily conserved zinc finger proteins present in many animal species. The orthology of the Sp genes in different animals is unclear and their evolutionary history is therefore controversially discussed. This is especially the case for the Sp gene *buttonhead *(*btd*) which plays a key role in head development in *Drosophila melanogaster*, and has been proposed to have originated by a recent gene duplication. The purpose of the presented study was to trace orthologs of *btd *in other insects and reconstruct the evolutionary history of the Sp genes within the metazoa.

**Results:**

We isolated Sp genes from representatives of a holometabolous insect (*Tribolium castaneum*), a hemimetabolous insect (*Oncopeltus fasciatus*), primitively wingless hexapods (*Folsomia candida *and *Thermobia domestica*), and an amphipod crustacean (*Parhyale hawaienis*). We supplemented this data set with data from fully sequenced animal genomes. We performed phylogenetic sequence analysis with the result that all Sp factors fall into three monophyletic clades. These clades are also supported by protein domain structure, gene expression, and chromosomal location. We show that clear orthologs of the *D. melanogaster btd *gene are present even in the basal insects, and that the *Sp5*-related genes in the genome sequence of several deuterostomes and the basal metazoans *Trichoplax adhaerens *and *Nematostella vectensis *are also orthologs of *btd*.

**Conclusions:**

All available data provide strong evidence for an ancestral cluster of three Sp-family genes as well as synteny of this Sp cluster and the Hox cluster. The ancestral Sp gene cluster already contained a *Sp5/btd *ortholog, which strongly suggests that *btd *is not the result of a recent gene duplication, but directly traces back to an ancestral gene already present in the metazoan ancestor.

## Background

Zinc finger transcription factors are a large and widespread family of DNA binding proteins and play an important role in transcriptional regulation (e.g. [1]). The general transcription factor Sp1 (named after the original purification method through sephacryl and phosphocellulose columns) was the first identified and cloned binding-specific human transcription factor [2-4]. In the meantime a number of additional genes related to *Sp1 *have been identified in the human genome, and homologous genes have been isolated from several other animal species as well (e.g. [1,5]). The members of this Sp-family of transcription factors share three highly conserved Cys2His2-type zinc fingers, which bind to G-rich DNA elements, such as GC-boxes (GGGGCGGGG) and GT/CACC-boxes (GGTGTGGGG) [4]. These binding sites are present in many control regions of both tissue-specific and ubiquitously expressed genes [6,7] indicating that Sp-family transcription factors potentially regulate a large number of target genes. Indeed, it was shown that Sp-family transcription factors have diverse functions throughout the embryonic development of humans and other animals. For instance, in vertebrates they are involved in cell cycle regulation, the control of morphogenetic pathways, the development of several organ systems, and they also have been linked to the development of cancer (e.g. [5,8-17]). In the fly *Drosophila melanogaster*, the gene *buttonhead *(*btd*) codes for a member of the Sp-family, which represents an important factor for the formation of several head segments and is also involved in the development of the central and peripheral nervous system [8,18-20].

The number of Sp-family genes present in the genome varies in the Metazoa. Humans and mice, for example, have nine Sp-family genes [5], and some teleost fishes have even more (11 in the pufferfish *Fugu rubripes *[21], 13 in the zebrafish *Danio rerio *[22]). From *D. melanogaster *two Sp-family genes have been reported, *btd *and *D-Sp1 *[8], but a third one is present in the fully sequenced genome sequence [23]. This variable complement of Sp-family genes and their evolutionary diversification made it difficult to assign orthology between the genes of different species. Therefore, the ancestral number of Sp-family genes and the evolution and orthology of the hitherto identified Sp-family genes was unclear. This situation also led to a considerable confusion in the nomenclature of the Sp-family genes and to several unfortunate designations of not directly homologous Sp-family members with homonymous names thus misleadingly suggesting orthology. For example, *D. melanogaster D-Sp1 *is not most closely related to human *Sp1 *but to *Sp8 *[24] and the gene originally termed mouse *mBtd *is in fact orthologous to *Sp8 *[13].

Especially the origin and orthology of the *D. melanogaster *head gap gene *btd *has been debated. Previous studies discovered functional similarities between *btd *and some vertebrate Sp genes, but could not confidently identify a genuine *btd *orthologue in vertebrates [13,15,25], and it had been proposed that the *btd *gene might be the result of a recent gene duplication when another Sp-family gene, *D-Sp1*, in the vicinity of *btd *was discovered in *D. melanogaster *[8,20]. This gene is not only located directly next to *btd*, but the two genes also have similar postblastodermal expression patterns and partially overlapping developmental functions [8,20]. All this suggested that *btd *evolved by a tandem duplication in the phylogenetic lineage leading to *D. melanogaster*.

In order to reconstruct the evolution of the Sp-family genes, we have first tried to trace homologs of *btd *in other insects. We have surveyed not only additional dipterans and other holometabolous insects, but we have also searched for Sp-family genes in representatives of hemimetabolous insects (the heteropteran *Oncopeltus fasciatus*) and the primitively wingless ectognathous and entognathous hexapods (the zygopteran *Thermobia domestica *and the collembolan *Folsomia candida*, respectively). We could identify clear orthologs of the *D. melanogaster btd *gene in these basal hexapods, indicating that the proposed gene duplication did not take place recently within the insects. We have therefore performed a comprehensive study of Sp-family gene evolution based on phylogenetic sequence analysis, protein domain structure characteristics, spatio-temporal mRNA expression analysis, as well as genomic localisation analysis. Our phylogenetic analysis shows that the available Sp-family factors fall into three large clades and that a true *btd *ortholog is already present in the basal metazoans *Trichoplax adhaerens *and *Nematostella vectensis*. The proteins in each clade also display similar structural characteristics and often form a cluster of three genes in the genome. Intriguingly, the available data suggest that this Sp gene cluster has been ancestrally linked to the Hox gene cluster and in the vertebrates appears to have been affected by the multiple duplications of this cluster. This synteny and co-evolution of the Hox and the Sp clusters in the vertebrates also explains the high number of Sp-family genes in this animal group.

## Results and Discussion

### A search for Sp-family genes in insects and crustaceans

As mentioned in the introduction, previous work had suggested that *D. melanogaster *possesses two closely related Sp genes, *btd *and *D-Sp1 *[8,19]. However, a search in the fully sequenced *D. melanogaster *genome revealed the presence of an additional gene, *CG5669*, with high similarity to *btd *and *D-Sp1*. This complement of three Sp-family genes could be the result of a recent gene duplication [8,20]. In order to identify when such a gene duplication event might have occured, we sought to identify the number of Sp-family genes in additional insect species.

We searched the genome sequence of selected insect species with fully sequenced genomes. In addition we performed PCR-based surveys in specially selected additional species. In the Diptera, a complement of three Sp-family genes seems to be the rule: in the genome sequences of *Drosophila pseudobscura *and the mosquito *Anopheles gambiae *we found three different Sp-family genes each. We then searched in the genomes of species outside the Diptera. In the lepidopteran *Bombyx mori *(silk moth), the hymenopterans *Apis mellifera *(honeybee) and *Nasonia vitripennis *(jewel wasp), and the coleopteran *Tribolium castaneum *(flour beetle) we also detected three Sp-family genes each. This taxon sampling included only holometabolous insects and we have therefore also isolated cDNA fragments of Sp-family genes from representatives of the hemimetabolous and the primitively wingless hexapods. In the higher hemimetabolous heteropteran *O. fasciatus *(milkweed bug), we were able to isolate two different Sp-family gene fragments. The Zygentoma represent the youngest branch of the primitively wingless insects [26]. We have used the zygentoman *T. domestica *(firebrat), from which we could isolate three different Sp-family gene fragments. The Collembola are members of the most basal branch of the primitive hexapods (Entognatha) [26]. In the collembolan *F. candida *(white springtail), we were also able to detect three different fragments of Sp-family genes.

These results show that a complement of three Sp-family genes is present in all studied hexapod species, except for *O. fasciatus *for which the genome sequence is not available and a third Sp-family member could have been missed in our PCR-based search. We have then tried to establish the number of Sp-family genes in the Crustacea, which phylogenetically is the sister group of the insects according to recent analyses (e.g. [27-30]). The waterflea *Daphnia pulex *is a member of the Branchiopoda. In the fully sequenced genome of *D. pulex *we detected the presence of three different Sp-family genes. The Malacostraca (higher crustaceans) are a group of primitively marine species. We have used PCR to isolate Sp-family gene fragments from the malacostracan *Parhyale hawaiensis *(beachhopper), which yielded two different fragments. However, as with the results for *O. fasciatus *the PCR survey may have missed an additional Sp-family gene in *P. hawaiensis*.

Taken together, these results strongly suggest that a complement of three different Sp-family genes is ancestral in the arthropods. Interestingly, three different Sp-family genes are also present in the fully sequenced genomes of the basal chordate lineage *Branchiostoma floridae*, and the echinoderm *Strongylocentrotus purpuratus*. Three different Sp-family genes are also present in the fully sequenced genomes of the cnidarian *N. vectensis*, and the placozoan *T. adhaerens *- both representing basal branches in the metazoan phylogenetic tree. This could be taken as evidence that the possession of three Sp-family genes is ancestral in the Metazoa. On the other hand, the high number of Sp-family genes in the genomes of vertebrates (e.g. nine Sp-family genes in humans and mice, 7 in the chicken, and more than 10 in fish), indicates that the Sp-genes can be subject to frequent duplications. Thus, the "triplets" in insects, cnidarians, placozoans, echinoderms, and basal chordates might potentially have originated independently.

### Phylogenetic analysis of Sp-family genes supports three large clades

**Figure 1:**
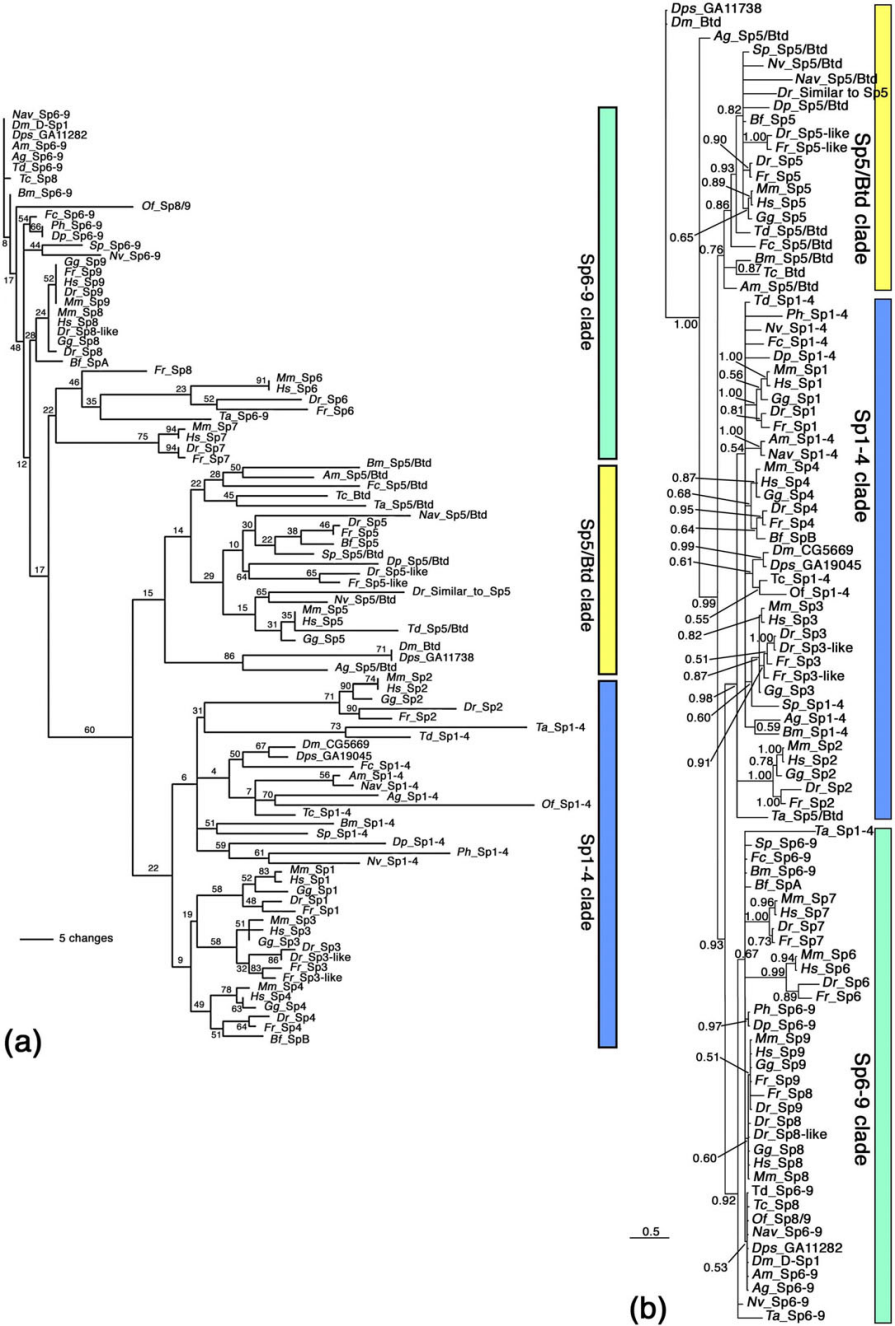
**Phylogenetic sequence analysis of Sp factors from diverse metazoan species**. The analysis reveals three large monophyletic clades (see text for details). (a) Unrooted majority rule consensus computed from 1000 intermediate trees produced with the Quartet Puzzling method [87]. The reliability values are given at the tree egdes. (b) Unrooted majority rule consensus Bayesian tree computed with the MR_BAYES program [83]. The posterior probability of the partitions is indicated by the branch. The abbreviations for the species names are given in the list of abbreviations. The sequence alignment is given in Additional File 1.

In order to distinguish between a possible ancestral set of three Sp-family genes and the alternative possibility of several independent duplication events, we reconstructed the evolutionary history of identified Sp-family factors and assigned orthology by phylogenetic sequence analysis. We used the amino acid sequence of the region including the Btd box, the three zinc fingers and the sequence in between these two domains of all available Sp-family factors of *Homo sapiens *(human), *Mus musculus *(mouse), *Gallus gallus *(chicken), *D. rerio *(zebrafish), *F. rubripes *(pufferfish), *B. floridae *(lancelet), *S. purpuratus *(sea urchin), *T. adhaerens *(placozoan), *N. vectensis *(sea anemone), and the insect and crustacean species mentioned above in a maximum likelihood analysis with the Tree Puzzle program package. The resulting unrooted tree is shown in Fig. 1a and the alignment is shown in Additional File 1. The tree comprises three large monophyletic groups. One clade contains Sp1, Sp2, Sp3 and Sp4 of the vertebrate species and a single Sp representative of each of the invertebrate species. We term this clade the Sp1-4 clade. The second clade contains Sp5 of the vertebrate species and again a single Sp representative of each of the invertebrate species, except for *O. fasciatus *and *P. hawaiensis *for which we failed to obtain three different Sp-family genes in our PCR survey. Because this clade also contains the well-known Btd from *D. melanogaster*, we call this clade the Sp5/Btd clade. The third clade contains Sp6, Sp7, Sp8, and Sp9 of all vertrebrate species and a single Sp representative of each of the invertebrates. We call this clade the Sp6-9 clade. In order to facilitate the unique identification of the genes, we refer to all genes (except those that already have an official name) using the clade name to which they belong in this phylogenetic analysis. The distribution of a single Sp factor of each invertebrate species to each of the three clades strongly suggests that a set of three Sp-family genes, namely one *Sp1-4*, one *Sp5/btd *and one *Sp6-9 *gene, is the ancestral state in the Metazoa and that the higher number in vertebrates resulted from further duplications in the vertebrate lineage due to the whole genome duplications that occured early in vertebrate evolution (discussed below).

We have in addition performed a Bayesian analysis of the same dataset. The resulting unrooted tree is shown in Fig. 1b. The Bayesian tree differs from the quartet puzzling tree in several places, but the only marked differences are Sp5/Btd and Sp1-4 from *T. adhaerens*, which are not included in the Sp5/Btd and Sp1-4 clade, respectively. The inconsistent placement of these *T. adhaerens *sequences in the two analyses might be explained by the phylogenetically old age of this lineage. Importantly, the three monophyletic clades Sp1-4, Sp5/Btd, and Sp6-9 are also recovered with this method and all three clades have very high support values. Thus, this additional analysis over all supports the quartet puzzling analysis.

### Protein structure supports the existence of two large groups of Sp factors

**Figure 2:**
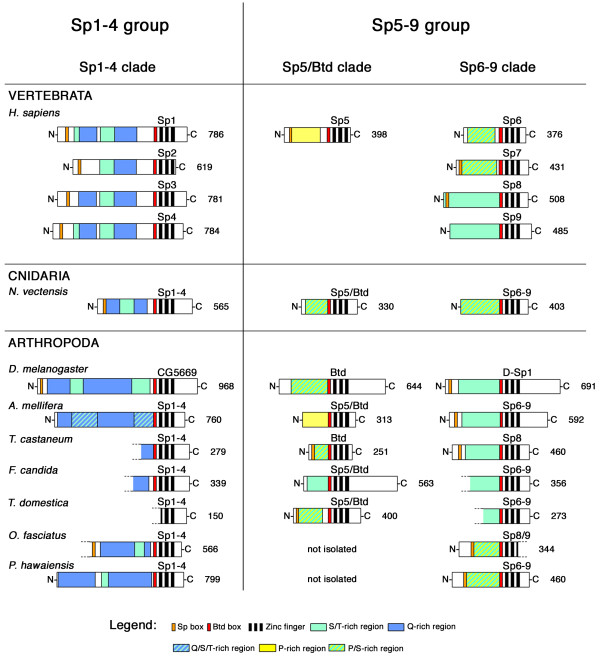
**Protein domain structure of selected Sp-family proteins**. The proteins are arranged into columns according to the clades obtained in the phylogenetic sequence analysis (Sp1-4 clade, Sp5/Btd clade, Sp6-9 clade). All proteins are oriented with their amino-terminus (N) to the left, and the carboxy-terminus (C) to the right. The length of each protein is given next to the C-terminus (number of amino acids), and the name of the protein is given above the protein. Incomplete termini of proteins are indicated by dashed lines. Structural domains are indicated by different colors and shadings explained in the legend below the proteins. Protein domain color coding after Bouwman and Philipsen [31].

It has been noted previously that the Sp proteins contain additional structural domains besides the zinc fingers and Btd box (e.g. [31]). A large portion of the N-terminal end of the proteins is enriched for certain amino acid residues. We have therefore compared the composition of Sp proteins from human, sea anemone, and selected arthropods (Fig. 2). The proteins of the Sp1-4 clade are longer proteins characterized by a (mostly) bipartite glutamine-rich region divided by a region enriched mostly for serine and threonine. These proteins form a well recognizable grouping that we call Sp1-4 group. The structure of the Sp1-4 group is clearly different from the Sp proteins of the Sp5/Btd and Sp6-9 clades (Fig. 2). These latter two clades contain shorter proteins (on average), and are more similar to each other than each is to the Sp1-4 group and we therefore group the two clades together in a grouping that we call Sp5-9/Btd group. The N-terminal end of these proteins contains only a single long region enriched for serine and/or proline. However, we note a trend in the Sp5/Btd clade towards the accumulation of more proline, whereas in the Sp6-9 clade there is a clear trend towards accumulating serine and threonine in the N-terminal portion. Thus, the protein structure data also support the existence of three different groups of Sp-factors, but suggest that the Sp5/Btd clade and the Sp6-9 clade are more closely related.

### Embryonic expression patterns of insect and crustacean Sp genes

All available data collectively and consistently suggest that a small Sp gene cluster comprising three Sp genes is ancestral in the Metazoa and that the triplets present in the insects derive from these ancestral three genes, i.e. the genes in the respective clades are orthologous. This argues against the alternative hypothesis that the sets of three Sp genes in the different insect species originated by independent duplication events. As an additional test of the orthologous nature of the three Sp genes in the different insect species we compared their expression patterns during embryogenesis by in situ hybridization. We reasoned that the genes of the same clade should show similar expression patterns in all species if they were true orthologs, but show different patterns if they originated through unrelated duplication events. In the following we compare the expression data from insects, the crustacean *P. hawaiensis *and published data from vertebrates arranged according to the three Sp-gene clades.

#### The genes of the Sp1-4 clade

**Figure 3:**
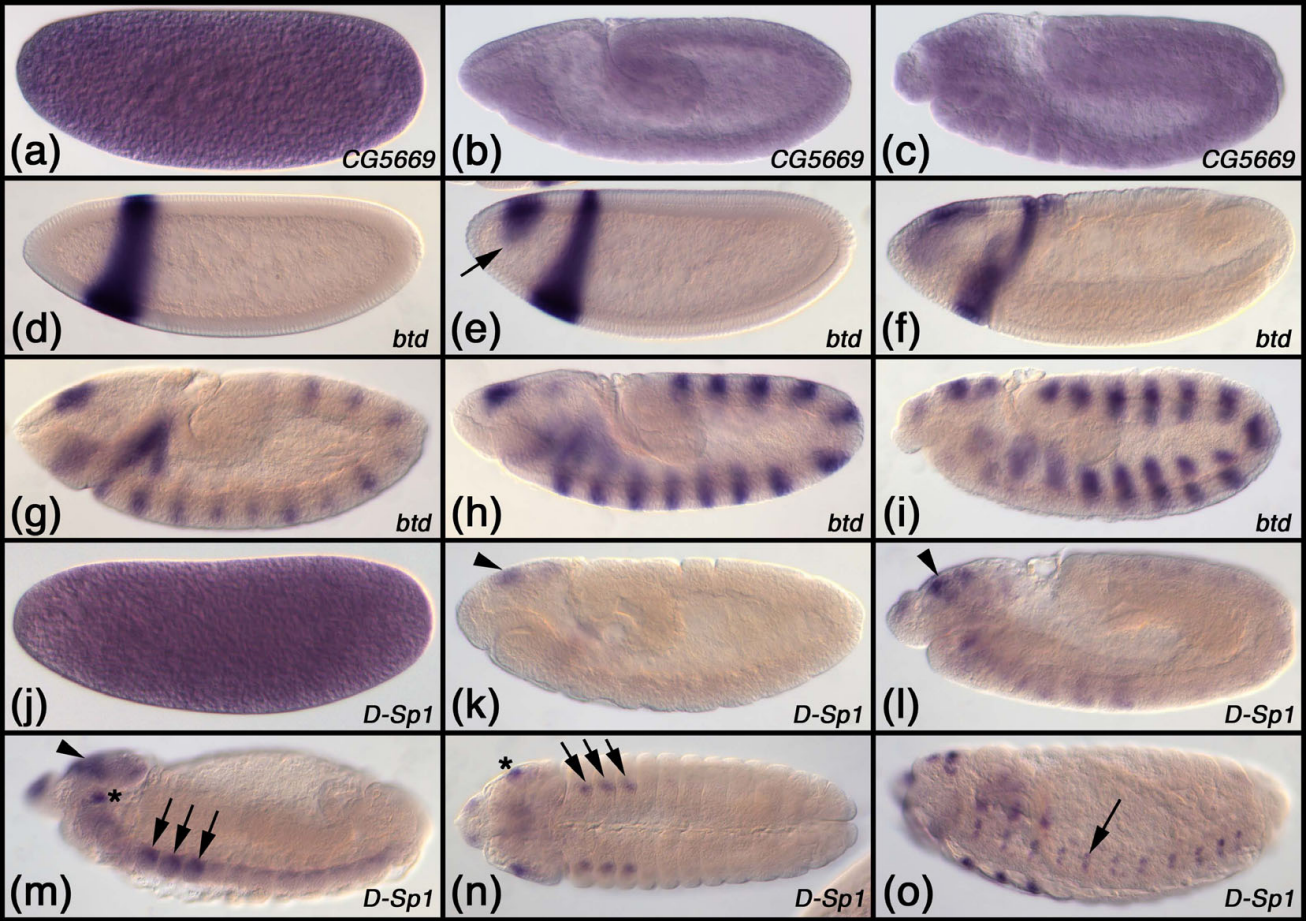
**Embryonic expression patterns of Sp genes in *Drosophila melanogaster***. (a-c) Expression of the *Sp1-4 *representative *CG5669*. (a) Stage 2 embryo. (b) Stage 9 embryo. (c) Stage 11 embryo. (d-i) Expression of the *Sp5/btd *representative *btd*. (d) Stage 4 embryo. (e) Stage 5 embryo. The arrow points to the anterior head domain that appears after the head stripe. (f) Stage 6 embryo. (g) Stage 8 embryo. (h) Stage 9 embryo. (i) Stage 11 embryo. (j-o) Expression of the *Sp6-9 *representative *D-Sp1*. (j) Stage 2 embryo. (k) Stage 10 embryo. (l) Stage 11 embryo. (m) Stage 13 embryo. (n) Stage 13 embryo, ventral view. (o) Stage 15 embryo. The arrowhead in k, l, m points to expression in the developing brain. The asterisk in m, n indicates expression in the antennal primordium. The arrows in m, n point to the thoracic limb primordia. The arrow in o points to expression in the developing ventral nerve cord. All embryos are arranged with anterior to the left.

**Figure 4:**
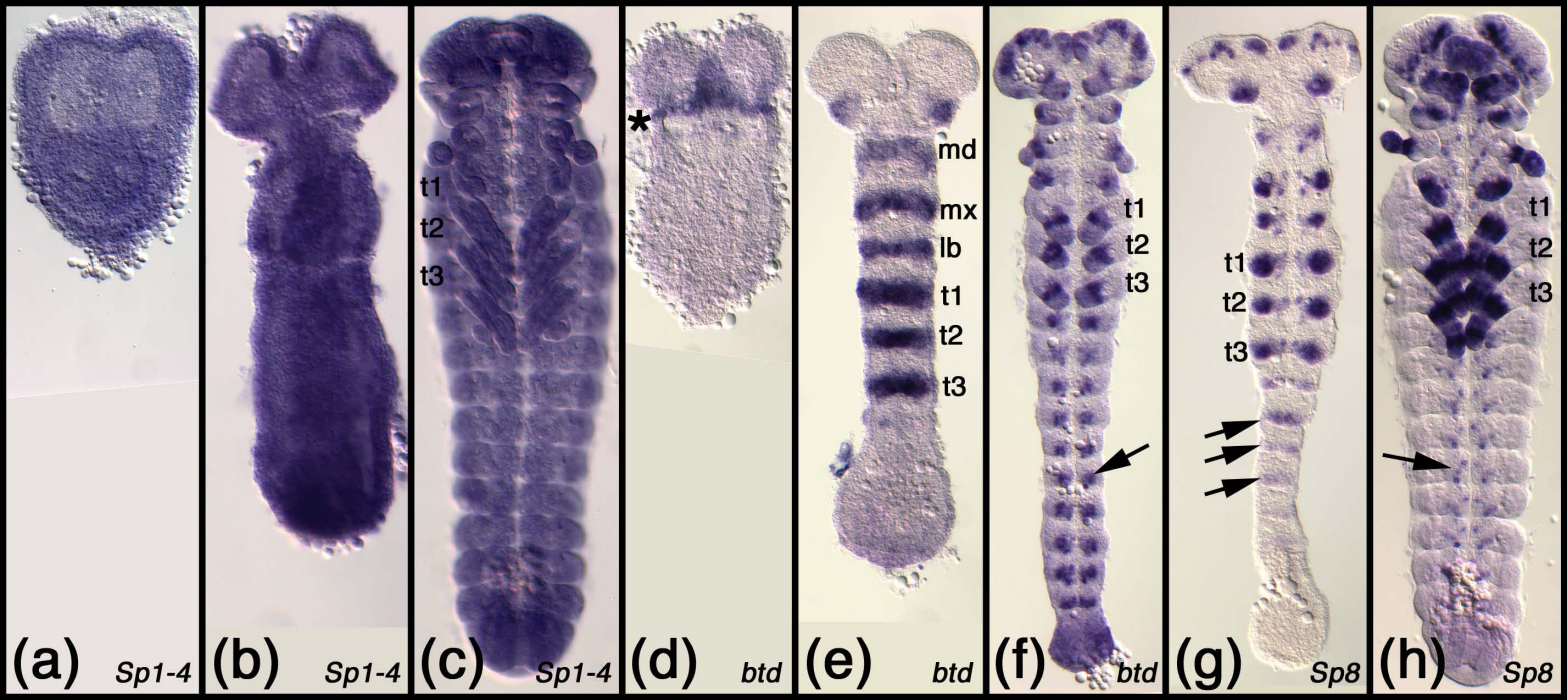
**Embryonic expression patterns of Sp genes in *Tribolium castaneum***. (a-c) Expression of the *Sp1-4 *representative at the gastrulating germband stage (a), after serosal closure (b) and at mid germband retraction (c). (d-f) Expression of the *Sp5/btd *representative *Tc-btd *shortly before serosal closure (d), at beginning germ band elongation (e), and beginning germ band retraction (f). The asterisk in d denotes the early head stripe expression domain. The arrow in f points to expression in the developing ventral nerve cord. (g, h) Expression of the *Sp6-9 *representative *Tc-Sp8 *at mid germband elongation (g) and at mid germband retraction (h). The arrows in g and h point to expression in the developing ventral nerve cord. All embryos are oriented with anterior to the top. Abbreviations: md, mandibulary segment; mx, maxillary segment; lb, labial segment; t1 - t3, thoracic segments 1 to 3.

**Figure 5:**
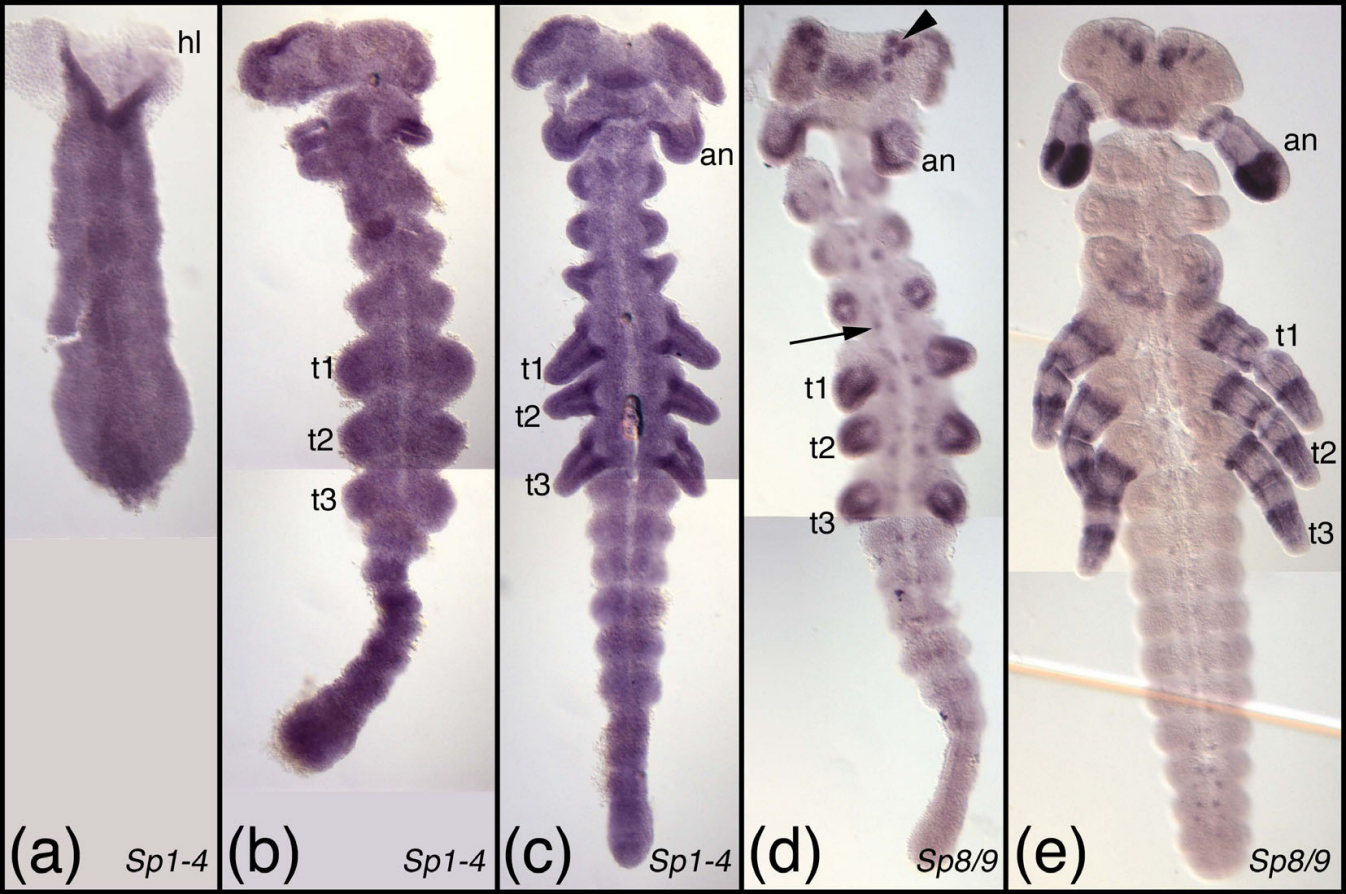
**Embryonic expression patterns of Sp genes in *Oncopeltus fasciatus***. (a-c) Expression of the *Sp1-4 *representative after serosal closure (a), at early germband elongation (b), and at full germband elongation (c). (d, e) Expression of the *Sp6-9 *representative *Of-Sp8/9 *at full germband elongation (d) and mid germband retraction (e). The arrowhead and arrow in d denote expression in the brain and ventral nervous system, respectively. All embryos are oriented with anterior to the top. Abbreviations see Fig. 4. Additional abbreviations: hl, head lobe; an, antennal segment/appendage.

**Figure 6:**
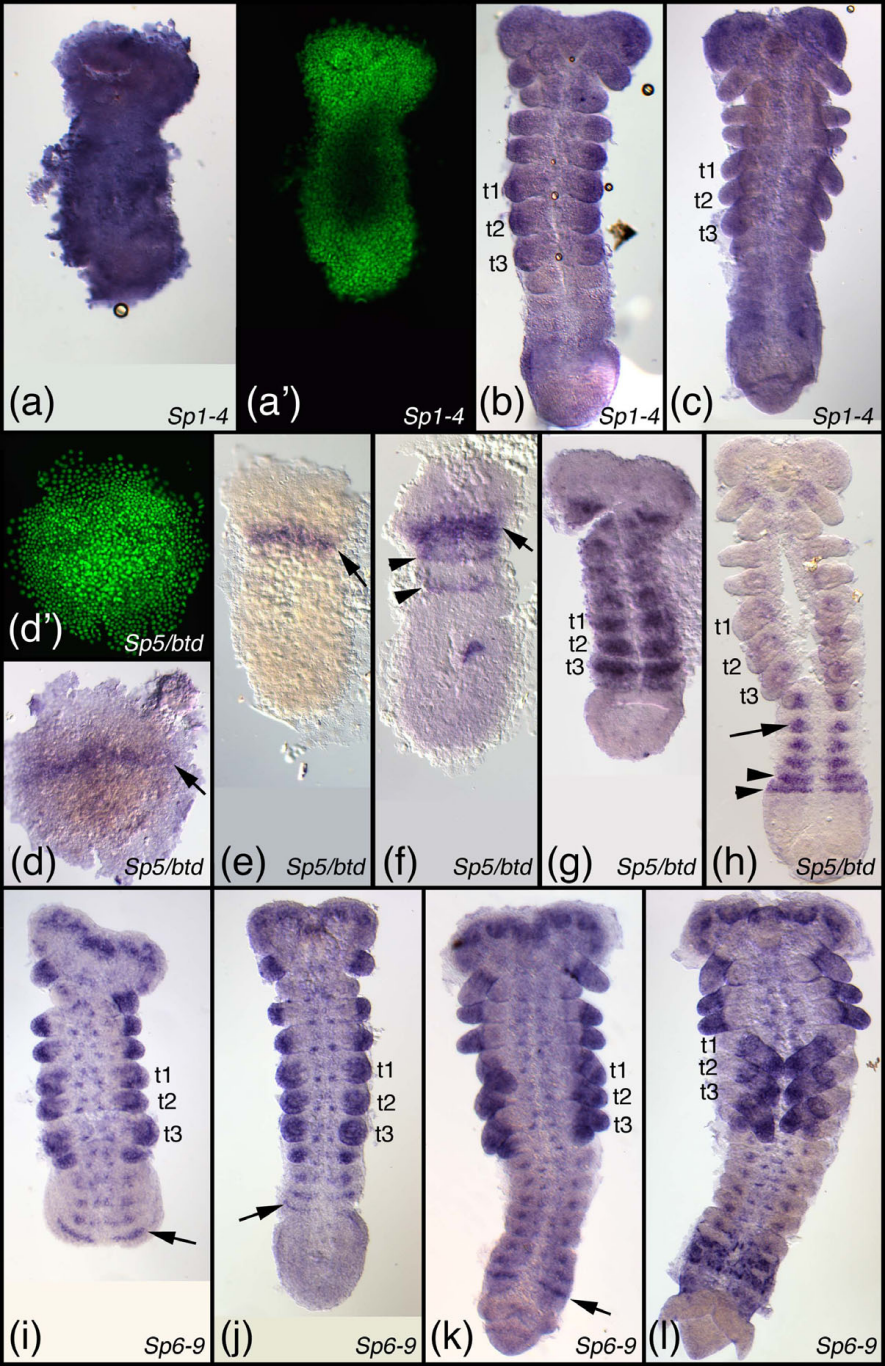
**Embryonic expression patterns of Sp genes in *Thermobia domestica***. (a-c) Expression of the *Sp1-4 *representative at the early germ band stage (a), mid germ band elongation (b), and late germ band elongation (a). (a') is the epifluorescence image (Sytox Green staining) of the embryo in a. (d-h) Expression of the *Sp5/btd *representative at the blastoderm stage (d), early germ band stage (e), starting germ band elongation (f), early germ band elongation (g), and late germ band elongation (h). The arrow in d-f points to the early head stripe. The arrowheads in f, h point to metameric stripes. The arrow in h points to expression in ventral nervous system. (d') is the epifluorescence image (Sytox Green staining) of the embryo in d. (i-l) Expression of the *Sp6-8 *representative at mid germ band elongation (i, j; the embryo in j is slightly more advanced than the one in i), early germ band retraction (k), and late germ band retraction (l). The arrow in i-k points to segmental stripes in young segments that just have formed from the posterior growth zone. All embryos are oriented with anterior to the top. Abbreviations see Fig. 4.

**Figure 7:**
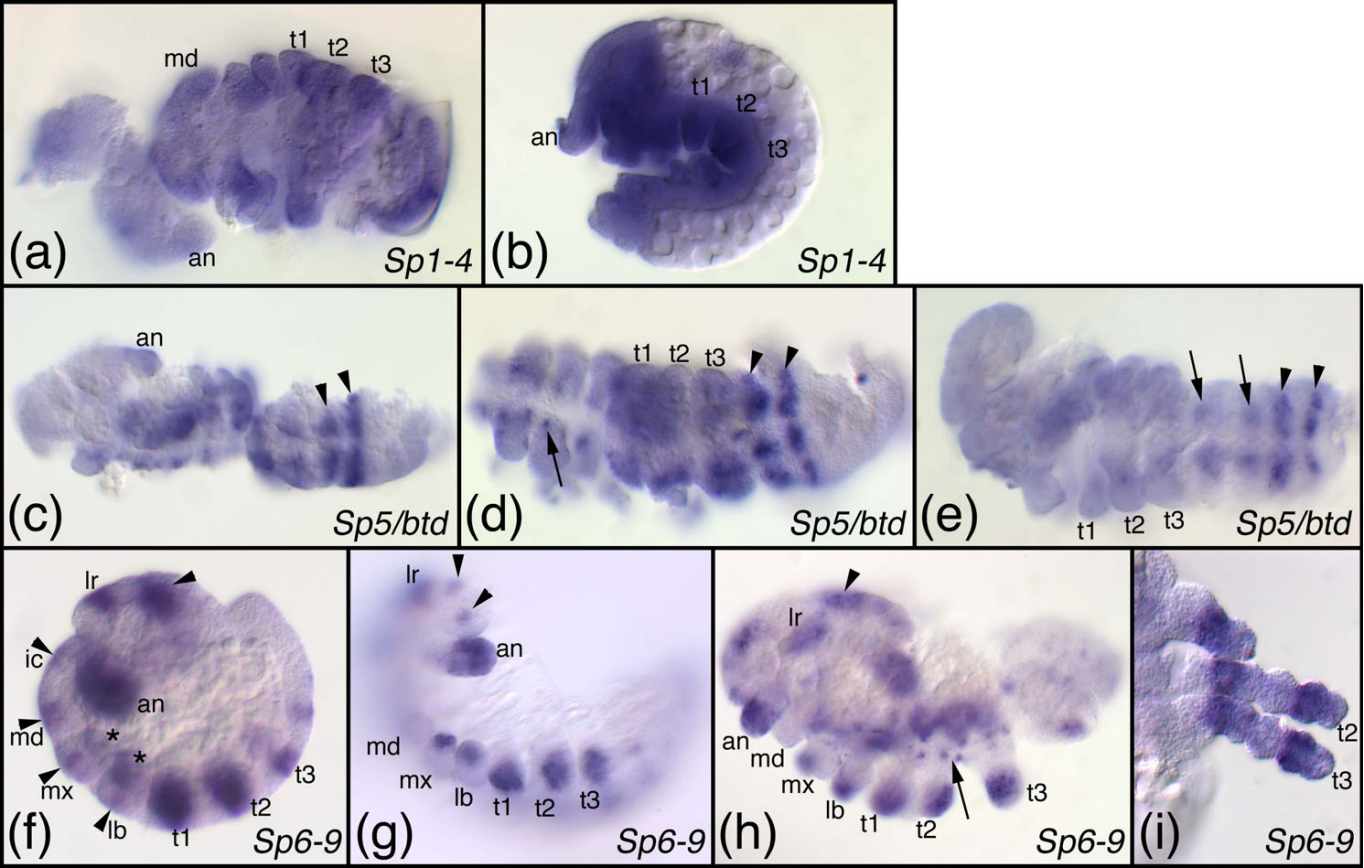
**Embryonic expression patterns of Sp genes in *Folsomia candida***. (a, b) Expression of the *Sp1-4 *representative at early germ band retraction (a) and beginning dorsal extension (b). (c-e) Expression of the *Sp5/btd *representative at mid germ band extension (c), late germ band extension (d, note that the anterior head has been lost during preparation), and early germ band retraction (e). The arrowheads in c-d point to metameric stripes. The arrows in d, e point to expression in the developing ventral nervous system. (f-i) Expression of the *Sp6-9 *representative at early germ band extension (f), mid germ band extension (g), and late germ band retraction (h). The asterisks in f denote expression in the limb buds of the maxillary and labial segment. The arrowheads in f-h point to expression spots in the brain and neural tissue of the head segments. The arrow in h points to punctate expression in the developing ventral nerve cord. (i) shows dissected legs of an embryo after dorsal closure. Note that the first thoracic leg has been damaged during preparation. Anterior is to the left in panels a-h. All panels show ventral aspects except for b, f, g which are lateral views. In i distal is to the right. Abbreviations see Fig. 4. Additional abbreviations: an, antennal segment/appendage; lr, labrum; ic, intercalary segment.

**Figure 8:**
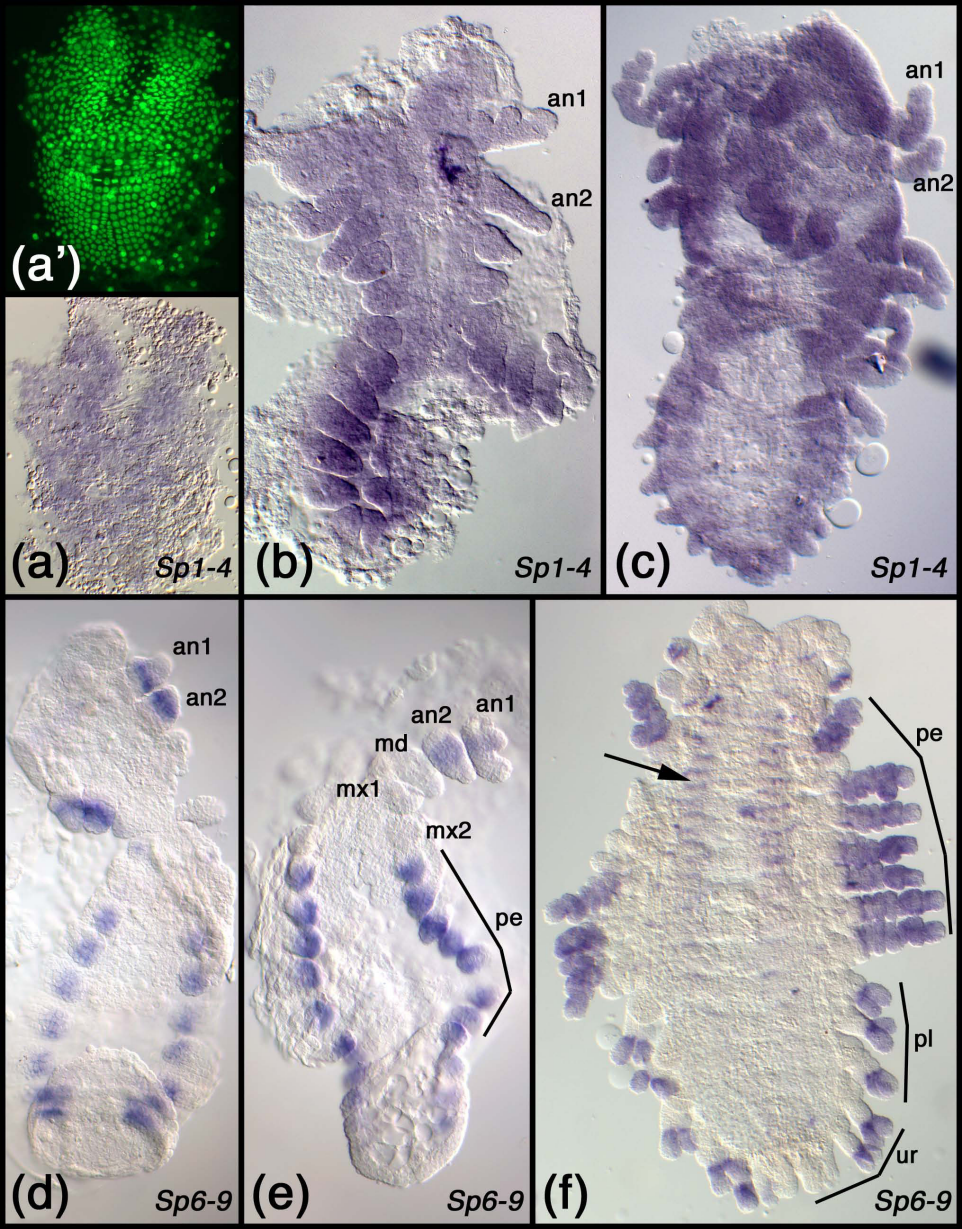
**Embryonic expression patterns of Sp genes in *Parhyale hawaiensis***. (a-c) Expression of the *Sp1-4 *representative at stage S12 (a), stage S19 (b), and stage S22 (c). Staging according to Browne et al. [68]. (a') is the epifluorescence image (Sytox Green staining) of the embryo in a. (d-f) Expression of the *Sp6-9 *representative at stage S17 (d), stage S18 (e), and stage S23 (f). The arrow in f points to expression in the ventral nervous system. Abbreviations: an1, first antenna; an2, second antenna; md, mandible; mx1, first maxilla; mx2, second maxilla; pe, peraeopods; pl, pleopods; ur, uropods.

*CG5669*, which is the *D. melanogaster *representative of this clade, is maternally contributed (Fig. 3a) and then expressed ubiquitously throughout development (Fig. 3b, c). In *T. castaneum *the *Sp1-4 *gene (in [32] previously termed *Tc-SP1234*) is expressed ubiquitously throughout development as well (Fig. 4a-c). The same is true for the *Sp1-4 *gene of *O. fasciatus *(Fig. 5a-c), *T. domestica *(Fig. 6a-c) and *F. candida *(Fig. 7a, b). In the crustacean *P. hawaiensis *the *Sp1-4 gene *is also expressed ubiquitously throughout all studied developmental stages (Fig. 8a-c). The members of this clade from the mouse have not all been studied as to their embryonic expression pattern, but data are available for murine *Sp1*, *Sp3 *and *Sp4 *[33-37]. All three genes are expressed ubiquitously during development. Taken together, these data show that all analyzed members of this clade are expressed in a similar ubiquitous fashion, strongly supporting the orthology of the genes.

#### The genes of the *Sp5/btd *clade

The expression of *btd *(the *D. melanogaster *representative of the *Sp5/btd *clade) has been reported previously [8,19]. The gene is first expressed in an anterior head stripe (Fig. 3d) and a dorsal spot appears slightly later (Fig. 3e). The head stripe is roughly located in the area of the intercalary and mandibulary segment and later abuts the cephalic furrow (Fig. 3f). Later a metameric (segmentally repeated) pattern emerges that might be correlated with segment formation and peripheral nervous system development (Fig. 3g-i) [8,20]. Furthermore, *Dm btd *is expressed in the imaginal discs of legs and antennae [8,38]. The expression of the *T. castaneum btd *gene has been published before [32] and is very similar to the *D. melanogaster btd *pattern: *Tc-btd *is expressed in an early head stripe in the area of the intercalary and mandibulary segment (Fig. 4d) and later a metameric pattern emerges (Fig. 4e). In older stages the gene is also expressed in the appendages and in the nervous system (Fig. 4f). The expression pattern of *Sp5/btd *in *T. domestica *is very similar to the *T. castaneum btd *pattern. In the early blastoderm the gene is expressed in an anterior stripe (Fig. 6d), that lies in the intercalary/mandibulary area in slightly more advanced germ band stage embryos (Fig. 6e). Later a metameric pattern emerges (Fig. 6f, g) and in older stages expression in the nervous system and, weakly, in the appendages is detected (Fig. 6 h). In *F. candida *we were not able to detect an early head stripe for *Sp5/btd*, because our fixation protocol did not allow us to fix blastoderm stages of this species. The later expression pattern of *Sp5/btd *in *F. candida *is very similar to the other insects: there is a metameric expression (Fig. 7c, d), a weak expression in the appendages (Fig. 7d), and expression in the nervous system (Fig. 7e).

There are 3 genes related to *Sp5 *in the zebrafish genome. *Sp5 *(also known as *bts1*) [25], *Sp5-like *(also known as *spr2*) [39] and *similar-to-Sp5*. *Sp5 *in zebrafish is expressed in a head stripe along the midbrain-hindbrain boundary, in the otic vesicles, diencephalon, tail bud, and in the somites [25]. Zebrafish *Sp5-like *expression is partially overlapping the *Sp5 *expression in ectodermal and mesodermal tissue, the brain, trunk neural crest cells, and somites [39]. Mouse *Sp5 *is also expressed in a head stripe at the midbrain-hindbrain boundary, in the primitive streak, and later in the tail bud, otic vesicles, limb buds, the developing central nervous system, somites and pharyngeal region [12,40]. In summary, the expression of the genes in this clade are highly similar in the insects and clear similarities also exist to the expression in the vertebrates. This again supports the orthology of the genes in this clade.

#### The genes of the *Sp6-8 *clade

The expression of *D-Sp1 *(the *D. melanogaster *representative of the *Sp6-9 *clade) has been published previously [8,20]. The gene is maternally contributed (Fig. 3j), and earliest embryonic expression is seen in the brain (Fig. 3k, l). Later, strong expression is seen in the limb primordia of the antennae and legs (Fig. 3m, n) and in a punctate pattern in the ventral nerve cord (Fig. 3o). The expression of the *T. castaneum Sp8 *gene has been reported earlier [24]. Like the *D. melanogaster D-Sp1 *gene, the *T. castaneum Sp8 *gene is expressed in the brain, ventral nerve cord, and the limb buds (Fig. 4g, h). In the growing legs the gene is expressed in a pattern comprising several rings (Fig. 4h) [24]. The gene *Sp8/9 *from *O. fas*ciatus has been published recently [41]. *Sp8/9 *is expressed in the brain, in a punctate pattern in the ventral nerve cord and in the limbs (Fig. 5d). Similar to the legs in older *T. castaneum *embryos, the *O. fasciatus Sp8/9 *gene is expressed in several rings in the legs (Fig. 5e). The *Sp6-9 *gene from *T. domestica *is expressed in the limb buds (Fig. 6i, j) and later in at least two rings in the legs (Fig. 6k, l). In young segments that have just separated from the growth zone there is a stripe of *Sp6-9 *expression and in older segments the gene is expressed in a punctate pattern in the ventral nerve cord. There is also an expression domain in the brain. In the springtail *F. candida *the *Sp6-9 *gene is expressed in the brain and in a punctate pattern in the ventral nervous system (Fig. 7f-h). The gene is also expressed in the limb buds (Fig. 7f-h) and at later stages in two separate rings in the legs (Fig. 7i). These data show that the embryonic expression pattern of the *Sp6-9 *representatives is very similar in all studied insect species. These similarities extend to the crustaceans as shown by *Sp6-9 *expression in *P. hawaiensis*. In this species the gene is expressed in the limb buds (Fig. 8d, e) and at later stages in the peraeopods and in the two branches of the pleopods and the first two pairs of uropods (Fig. 8f). In addition, there is a punctate expression pattern in the ventral nerve cord (Fig. 8f).

Expression data for the members of this clade are also available from vertebrates. Intitial RT-PCR analysis of mouse *Sp6 *expression suggested expression in all tissues studied [42], but later studies showed a specific expression pattern in hair follicles and the apical ectodermal ridge (AER) of the developing limbs [15,43]. Consequently, *Sp6 *null mice are nude and show defects in skin, teeth, limbs (syndactyly and oligodactyly), and lung alveols. *Sp7 *(also known as *osterix*) is so far only documented to be expressed in the osteoblasts. Bone formation fails in *Sp7 *deficient mice due to impaired osteoblast differentiation [44-46]. Apart from expression domains in the nervous system (brain) both *Sp8 *and *Sp9 *are predominantly expressed in the AER of the limbs in mouse, chick and zebrafish and are essential for limb and fin outgrowth [13,14,47,48]. In summary, the expression patterns of the genes in this clade are strikingly similar in the insects and crustaceans and very similar expression patterns also exist from some vertebrate representatives of this clade, again supporting the orthology of the genes in this clade.

Summarizing the available gene expression data it is evident that the gene expression profiles of the arthropod and vertebrate members within each clade are very similar. This lends further support to our notion that the Sp-family genes in the Metazoa fall into three monophyletic clades that each derives from a single ancestral gene from a cluster comprising three genes. The ubiquitous pattern of the *Sp1-4 *genes separates them from the *Sp5/btd *and *Sp6-9 *genes that display more complex expression patterns frequently comprising at least domains in the nervous system, limbs and segments. This observation fully agrees with our analysis of protein structure that also suggests that the *Sp5/btd *clade and the *Sp6-9 *clade form a larger grouping (the *Sp5-9 *group).

### Chromosomal location of Sp genes suggest an ancestral triplet

**Figure 9:**
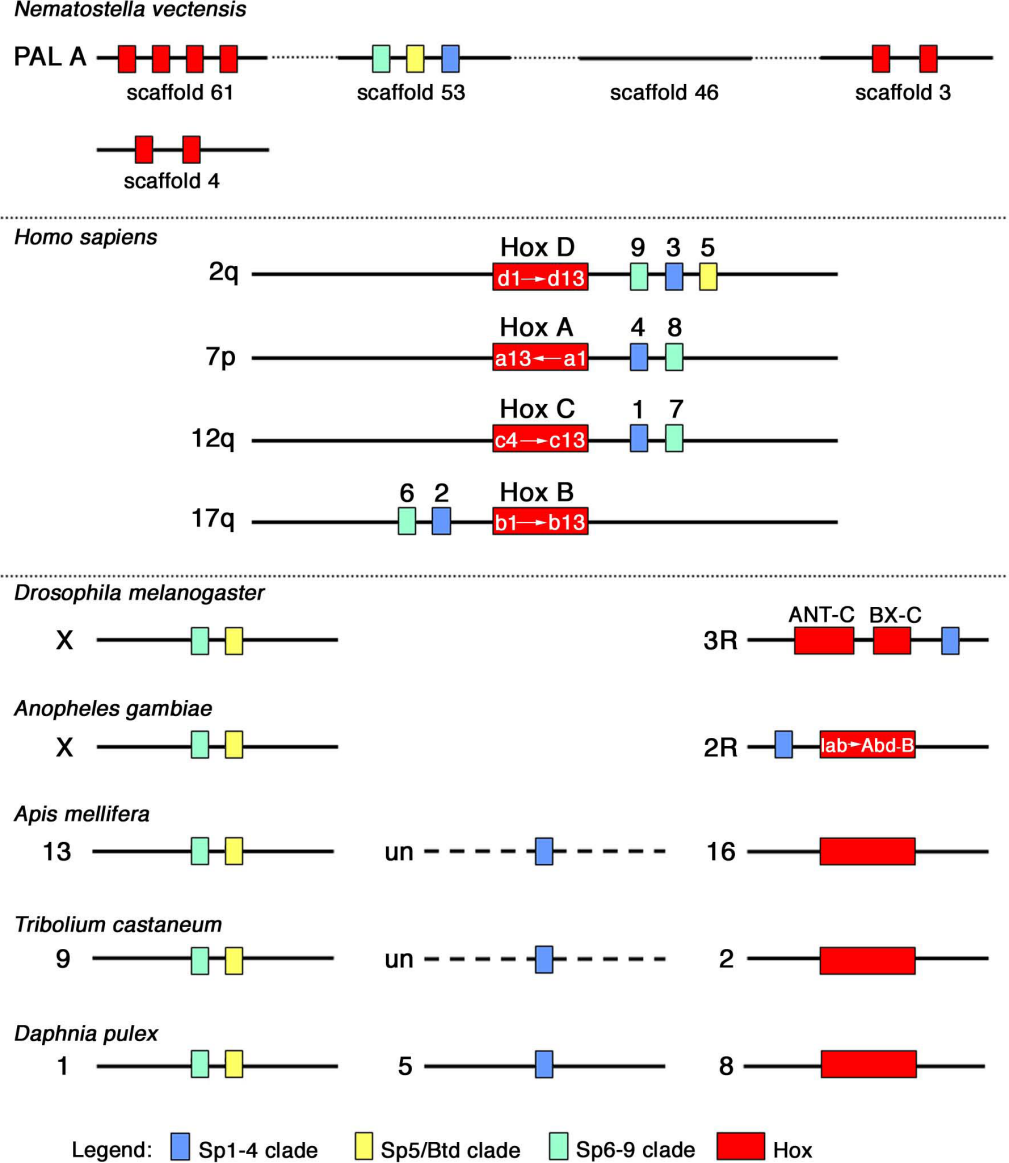
**Chromosomal location of the Sp-family genes in the genomes of selected animal species**. Single genes are represented by small colored boxes, Hox gene clusters are denoted by a larger red box. The Sp-family genes are color-coded according to the three clades revealed by the phylogenetic sequence analysis; the colors are explained in the legend below the drawings. The Hox cluster in *D. melanogaster *is split into two parts, the Antennapedia complex (ANT-C) and the Bithorax complex (BX-C). Continuous genomic regions are indicated by solid black lines, dashed lines indicate unclear conditions due to incomplete genome assembly. Genes, gene clusters and genomic loci are not drawn to scale. The numbers above the Sp genes in *H. sapiens *denote the gene name (*Sp1 *to *Sp9*). The denominations left of the black lines indicate the chromosome (X is the X-chromosome), or the linkage group (for *A. mellifera*, *T. castaneum*); un denotes localisation of the gene in an unassembled region of the genome in species where the genome assembly is incomplete. For *N. vectensis *the scaffolds containing Hox and Sp genes are shown and are arranged into the hypothetical ancestral linkage group PAL A [50]. The hypothetical linkage between these scaffolds is indicated by the dotted lines.

We have also established the location of the Sp-family genes in the genomes of fully sequenced and sufficiently annotated metazoan species; a schematic overview is shown in Fig. 9 and the exact locations are given in Additional File 2. Intriguingly, in the basal metazoan *N. vectensis *all three Sp-family genes are located next to each other on a single scaffold (scaffold 53). This situation is fully compatible with the notion that a triplet consisting of one Sp1-4, one Sp5/Btd, and one Sp6-9 gene is ancestral in the Metazoa. The close proximity of the genes on a single scaffold suggests that the Sp-family genes form a gene cluster of closely related genes evolved by tandem gene duplication similar to the genes in the Hox gene cluster. Ryan et al. [49] and Putnam et al. [50] have used the scaffold data of *N. vectensis *to reconstruct ancestral metazoan linkage groups (a kind of "ur-chromosomes"). Interestingly, the Sp cluster of *N. vectensis *is located next to the majority of the *N. vectensis *Hox genes on the hypothetical ancestral linkage group PAL A (Fig. 9, top) [50]. Only the two Hox genes on scaffold 4 are not included in the PAL A. This suggests that the Sp gene cluster and the Hox gene cluster were ancestrally located next to each other and might have conserved their close linkage in Cnidaria and vertebrates, and to a lesser extent in arthropods (Fig. 9). The Sp genes are located close to the Hox gene cluster in other animals as well (see also [31,51]. Intriguingly, in humans, a triplet of one *Sp1-4*, one *Sp5/btd*, and one *Sp6-9 *gene, namely *Sp3*, *Sp5*, and *Sp9*, is linked to the Hox D cluster and the remaining human Sp genes are arranged in duplets of one *Sp1-4 *and one *Sp6-9 *gene, which are linked to the remaining 3 Hox clusters, respectively (Fig. 9, center). In *D. melanogaster *and *A. gambiae *only the *Sp6-9 *clade gene is linked to the Hox gene cluster, while the remaining two genes are located close to each other on the X chromosome (Fig. 9, bottom). These two genes are also located close to each other on another chromosome than the Hox gene cluster in *A. mellifera*, *T. castaneum *and the crustacean *D. pulex*. In addition, the *Sp1-4 *gene representative is also not linked to the Hox cluster, although this is not fully established for *A. mellifera *and *T. castaneum*, because the *Sp1-4 *gene is annotated within unassembled reads not placed to the assembled chromosome. The genomes of *S. purpuratus*, *B. floridae *and *T. adhaerens *are not assembled to the chromosome or linkage group level, but preliminary analysis provided additional evidence for Sp-family gene clustering in these species as well. In *S. purpuratus *the *Sp1-4 *and *Sp5/btd *genes are located on the same scaffold. In both *B. floridae *(see also [52]) and *T. adhaerens *the *Sp5/Btd *and *Sp6-9 *genes are located on the same scaffold (see also [53]). Whether the Sp-family genes are also linked to the Hox genes in *S. purpuratus *(see [54]), *B. floridae *(see [55,56]), or *T. adhaerens *(see [57]) has to await the full assembly of the scaffolds.

## Conclusions

**Figure 10:**
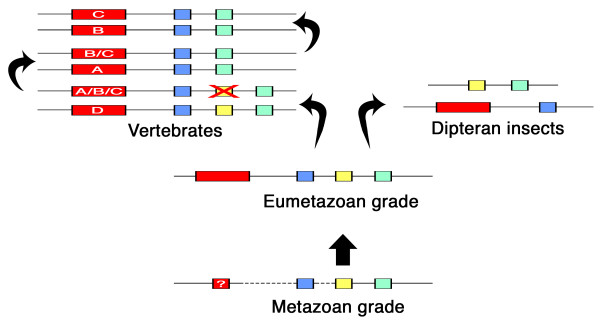
**Evolution and orthology of the Sp genes in the Metazoa**. The ancestral state in the Metazoa (metazoan grade) is hypothesized to have been a cluster of three Sp genes (one of each clade, color coded as in Fig. 9) linked to a single Hox gene (small red box; no Hox cluster is present yet). This is consistent with the data from *T. adhaerens*. Linkage data for the *Sp1-4 *gene, however, are missing and the nature of *Trox2 *gene is debated (indicated by the dashed lines and the question mark). In addition, data from the most basal metazoan group, the Porifera, are not yet available. The Sp gene cluster is conserved in the Eumetazoa (eumetazoan grade) and is linked to the Hox cluster. This is consistent with data from *N. vectensis*, and is further supported by comparative genomics [50]. Further evolution in the Vertebrata lineage lead to the multiplication of the Sp gene cluster along with the Hox gene cluster. The number of Sp gene clusters, their chromosomal location and Sp gene complement is fully compatible with the sequence (D(A(C, B))) of vertebrate Hox gene cluster duplication proposed by Bailey et al. [88]. We propose that the ancestral Sp gene cluster was duplicated, the duplicate lost the *Sp5/btd *gene, and this reduced cluster served as template for two additional Hox/Sp duplications. In the Insecta lineage the ancestral linkage of the Sp cluster with the Hox cluster was partially disbanded by the relocation of the *Sp5/btd *and *Sp6-9 *genes.

All available data suggest that a set of three Sp-family genes comprising one *Sp1-4 *gene, one *Sp5/btd*, and one *Sp6-9 *gene, is ancestral in the Metazoa (Fig. 10). No data are yet available from the most basal metazoan group, the Porifera (sponges), but at least two Sp-family genes are linked in the basal metazoan *T. adhaerens*. This can serve as evidence that the Sp-family triplet formed a small gene cluster already in the basal metazoan (Fig. 10, "metazoan grade"), but it is unclear whether this Sp gene cluster was initially linked to the Hox gene cluster. It is still debated whether *T. adhaerens *has any true Hox genes since it has only one Hox-like gene (*Trox-2*) along with one further, very derived gene with potential Hox-like affinities [57,58]. But it is yet unclear whether the single *T. adhaerens *Hox-like gene is physically close to the Sp-family genes.

The eumetazoan ancestor already possessed a triplet cluster of Sp-family genes (Fig. 10, "eumetazoa grade") as evidenced by the three closely linked Sp genes in the genome of the sea anemone *N. vectensis*. This cnidarian species has eight Hox genes. It is debated whether the Cnidaria represent a grade before or after the formation of a true Hox gene cluster, but recent analyses strongly suggest that the ancestral Cnidarian had indeed a genuine Hox gene cluster comprising at least one anterior and one posterior Hox gene [49,59]. This cluster apparently has broken apart during cnidarian evolution leading to the dispersed set of 8 Hox genes in *N. vectensis *[49]. None of these Hox genes in *N. vectensis *is on the scaffold that contains the Sp genes, but comparative genomics studies suggest that the four clustered Hox genes and the Sp gene cluster are located next to each other on the so called "PAL A" linkage group [50]. Thus, the Eumetazoa ancestor likely possessed a Sp gene cluster linked to the primordial Hox gene cluster (Fig. 10, "eumetazoan grade").

In the Bilateria the Hox cluster underwent further elaboration by gene duplications, whereas the nearby Sp gene cluster preserved the ancestral number of three genes. Nevertheless, the evolution of the Hox cluster also had an impact on the evolution of the Sp cluster in different ways in different bilaterian lineages. In the insects for example, the Sp gene cluster became partially independent from the Hox gene cluster by the relocation of the *Sp5/btd *and the *Sp6-9 *genes (Fig. 10, top right). In the dipterans the *Sp1-4 *gene is still linked to the Hox gene cluster, but in other insects (and in the crustacean *D. pulex*) the *Sp1-4 *gene appears to have become detached from the Hox gene cluster as well. In the vertebrates, the Hox gene cluster was duplicated several times leading to a total set of four Hox gene clusters in tetrapods [60], and the nearby Sp gene cluster evidently was duplicated along with the Hox gene cluster (Fig. 10, top left). Additional partial genome duplications have occurred in the teleost fishes [61,62] likely accounting for the additional Sp genes (e.g. in *D. rerio *and *F. rubripes*). In summary, our results show that the *btd *gene did not originate from a recent gene duplication, but traces back to an ancient *Sp5/Btd *gene already present in basal metazoans.

## Materials and methods

### Arthropod husbandry, embryo collection and fixation

The *O. fasciatus *(milkweed bug) culture was kept as described in Hughes and Kaufman [63]. Embryos of all stages were fixed as reported previously [64]. Dissections of milkweed bug embryos were performed under a fluorescence stereomicroscope using SYTOX Green nucleic acid stain (Invitrogen) before in situ staining [65]. *T. domestica *(firebrat) were cultured as described in Rogers et al. [66] with some modifications: Firebrats were kept in plastic containers in an incubator at 36°C and fed with oatmeal. For better handling especially of very young embryos during the dissection procedure, firebrat eggs were first boiled for 1 min in a waterbath and cooled on ice for 1 min. Afterwards, embryos were fixed for 1 h in fixative (4% formaldehyde in phosphate buffered saline and 0,1% Tween-20). Embryos were stained with SYTOX Green nucleic acid stain and dissected as described for *O. fasciatus *[65]. *F. candida *(white springtail) were raised at room temperature in plastic containers with a thin layer of plaster mixed with charcoal. Springtail embryos from 0-5 days were collected with a fine brush and put into a 1,5 ml reaction tube filled with 500 μl water. Embryos were boiled for 1 min in a waterbath, cooled on ice for 1 min, then put into a 50 μm mesh net and treated with 50% bleach for 6 min. Afterwards, embryos were washed with water and put into 100% Methanol. These embryos were then sonicated for 45 sec in Methanol, vortexed several times and stored at -20°C until use. *P. hawaiensis *(amphipod beachhopper) were cultured in shallow plastic boxes at 26°C filled with a thin layer of crushed coral substrate and artificial seawater (30 g/l of synthetic sea salt) and fed with dry fish flakes twice a week. Membrane pumps ventilated the water. Gravid amphipod females were anaesthesized with clove oil (10 μl per 50 ml seawater) and embryos were collected out of the brood prouch with forceps. Dissection and fixation was performed as described in Browne et al. [67].

### Gene cloning and sequence analysis

*D. melanogaster *embryos from 0-20 h, *T. castaneum *embryos from 0-72 h, *O. fasciatus *embryos from 0-96 h, *T. domestica *and *F. candida *embryos from 0-5 days, and *P. hawaiensis *embryos of all described stages [68], were used for mRNA isolation using the MicroPoly(A)Purist kit (Ambion). Double-stranded (ds) cDNA and RACE template synthesis was performed using the SMART PCR cDNA Synthesis kit and SMART RACE cDNA Amplification Kit (Clontech). Degenerate primers were designed based on alignments of differerent Sp factor sequences (e.g. *D. melanogaster*, *T. castaneum*, mouse). Sp factors of the different arthropod species used in this study were isolated with different combinations of the following degenerate primers: Fw_GRATCDCPNC (GGC MGG GCI ACI TGY GAY TGY CCI AAY TG), Fw_RCRCPNC (MGI TGY MGI TGY CCI AAY TG), Fw_CHV/IPGCGK (TGY CAY RTI CCI GGI TGY GGI AA), Rev_RSDELQRH (TGI CKY TGI ARY TCR TCI SWI C), Rev_KRFMRSDHL (ARR TGR TCI SWI CKC ATR AAI CKY AA). RACE PCR was performed with gene specific primers designed on the basis of the results of the degenerate primers PCR. RACE primer sequences are given in Additional File 3. PCR fragments were cloned into the pCR-II (Invitrogen) and sequenced. All newly isolated sequences have been submitted to the EMBL Nucleotide Database with the following accession numbers: *Of*_*Sp1-4 *[EMBL: FN562984]*, Td*_*Sp1-4 *[EMBL: FN562988], *Td*_*Sp5/btd *[EMBL: FN562989], *Td*_*Sp6-9 *[EMBL: FN562990], *Fc*_*Sp1-4 *[EMBL: FN562985], *Fc*_*Sp5/btd *[EMBL: FN562986], *Fc_Sp6-9 *[EMBL: FN562987], *Ph*_*Sp1-4 *[EMBL: FN562991]*, Ph*_*Sp6-9 *[EMBL: FN562992]. BLAST analysis was used to identify the *Sp1-4 *homologue of *D. melanogaster *and *T. castaneum*. Gene specific primers were made to amplify *Tc*_*btd *[GenBank: NM_001114320.1], *Tc*_*Sp8 *[GenBank: NM_001039420] and *Tc*_*Sp1-4 *[GenBank: XM_967159] from *T. castaneum *cDNA, as well as *Dm_btd *[GenBank: NM_078545], *Dm_D-Sp1 *[GenBank: NM_132351] and *Dm_CG5669 (Sp1-4) *[GenBank: NM_142975] from *D. melanogaster *cDNA. The sequences of these primers are given in Additional File 3. We have used the publicly available genome sequencing data for a selection of metazoan species: *H. sapiens *(Genome Reference Consortium Human Build 37 (GRCh37), Primary_Assembly) [69,70], *M. musculus *(Reference assembly (C57BL/6J)) [71], *N. vitripennis *[72], *D. melanogaster *(release 5.10) [23], *D. pseudobscura *(release Dpse_2.0) [73], *A. mellifera *(Amel_4.0) [74], *A. gambiae *(AgamP3.3) [75], *T. castaneum *(Tcas_3.0) [76], *B. mori *(version 01 BABH01000000) [77], *D. pulex *(JGI-2006-09) [78], *S. purpuratus *(Build 1.1) [79], *N. vectensis *(Nematostella vectensis v1.0) [50], *G. gallus *(Gallus_gallus-2.1) [80], *F. rubripes *(Fourth Fugu Genome assembly) [21], *D. rerio *[22], *B. floridae *(Branchiostoma floridae v1.0) [81], and *T. adhaerens *(Trichoplax adhaerens Grell-BS-1999 v1.0) [58]. Phylogenetic analysis of different Sp transcription factor sequences with the Quartet Puzzling method was performed as described in Prpic et al. [82]. Additional Bayesian analysis was performed using MrBayes [83] and the tree was visualized with PhyloWidget [84]. The accession numbers and the protein sequences alignment are described in Additional File 1.

### In situ hybridization

The length of the templates, the clone ID, and the RNA polymerase used for digoxygenin labeled RNA probe synthesis are given in Additional File 3. *D. melanogaster *and *T. castaneum in situ *was performed essentially as described in Wohlfrom et al. [85], *O. fasciatus in situ *hybridization was done according to Liu and Kaufman [64], *P. hawaiensis in situ *was performed as reported in Browne et al. [67], and *in situ *hybridizations for *F. candida *and *T. domestica *were done essentially as described in Hughes et al. [86].

## Abbreviations

The abbreviations used to denominate the different species are as follows: *Ag*: *Anopheles gambiae*; *Am*: *Apis mellifera*; *Bf*: *Branchiostoma floridae*; *Bm*: *Bombyx mori*; *Dm*: *Drosophila melanogaster*; *Dp*: *Daphnia pulex*; *Dps*: *Drosophila pseudobscura*; *Dr*: *Danio rerio*; *Fc*: *Folsomia candida*; *Fr*: *Fugu rubripes*; *Gg*: *Gallus gallus*; *Hs*: *Homo sapiens*; *Mm*: *Mus musculus*; *Nav*: *Nasonia vitripennis*; *Nv*: *Nematostella vectensis*; *Of*: *Oncopeltus fasciatus*; *Ph*: *Parhyale hawaiensis*; *Sp*: *Strongylocentrotus purpuratus*; *Ta*: *Trichoplax adhaerens*; *Tc*: *Tribolium castaneum*; *Td*: *Thermobia domestica*.

## Authors' contributions

NDS carried out the molecular genetic studies, genome location, protein domain analyses, embryological work, and drafted the manuscript. NMP performed the phylogenetic sequence analysis and helped to draft the manuscript. EAW and NMP helped in the analysis of the data and participated in the design and coordination of the study. EAW conceived of the study. All authors read and approved the final manuscript.

## Supplementary Material

Additional file 1 **Sequence alignment used as basis for the phylogenetic analysis shown in Fig**. 1. CLUSTAL X (1.81) multiple sequence alignment of different Sp factors comprising the conserved region of the Btd box (in blue) and the three zinc fingers (in red). Accession numbers of used Sp proteins: *Dm_*CG5669 [GenBank: NP_651232], *Dm_*Btd [GenBank: NP_511100], *Dm_*D-Sp1 [GenBank: NP_572579], *Dps_*GA19045 [GenBank: XP_001358829], *Dps_*GA22354 [GenBank: XP_002134535], *Dps*_GA12282 [GenBank: XP_001354397], *Ag*_Sp1-4 [GenBank: NZ_AAAB02008898], *Ag*_Sp5/Btd [GenBank: NZ_AAAB02008847], *Ag *Sp6-9 [GenBank: NZ_AAAB01008847]; *Nav*_Sp1-4 [GenBank: XP_001599101], *Nav*_Sp5/Btd [GenBank: AAZX01008599], *Nav*_Sp6-9 [GenBank: XP_001606079], *Am*_Sp1-4 [GenBank: XP_624316.2], *Am*_Sp5/Btd [GenBank: XP_001119912], *Am*_Sp6-9 [GenBank: XP_624528], *Bm*_Sp1-4 [GenBank: BABH01010251], *Bm*_Sp5/Btd [GenBank: BABH01024462], *Bm*_Sp6-9 [GenBank: AADK01002198], *Tc*_Sp1-4 [GenBank: XP_972252], *Tc*_Btd [GenBank: NP_001107792], *Tc*_Sp8 [GenBank: NP_001034509], *Of*_Sp8/9 [EMBL: FN396612], *Nv*_Sp1-4 [GenBank: XP_001635004], *Nv*_Sp5/Btd [GenBank: XP_001635002], *Nv*_Sp6-9 [GenBank: XP_001634948], *Sp*_Sp1-4 [GenBank: XR_025838], *Sp*_Sp5/Btd [GenBank: XP_789110.1], *Sp*_Sp6-9 [GenBank: XP_793203.2], *Hs*_Sp1 [GenBank: NP_612482], *Hs*_Sp2 [GenBank: NP_003101], *Hs*_Sp3 [GenBank: NP_003102], *Hs*_Sp4 [GenBank: NP_003103], *Hs*_Sp5 [GenBank: NP_001003845], *Hs*_Sp6 [GenBank: NP_954871], *Hs*_Sp7 [GenBank: NP_690599], *Hs*_Sp8 [GenBank: NP_874359], *Hs*_Sp9 [GenBank: NP_001138722], *Mm*_Sp1 [GenBank: NP_038700], *Mm*_Sp2 [GenBank: NP_084496], *Mm*_Sp3 [GenBank: NP_035580], *Mm*_Sp4 [GenBank: NP_033265], *Mm*_Sp5 [GenBank: NP_071880], *Mm*_Sp6 [GenBank: NP_112460], *Mm*_Sp7 [GenBank: NP_569725], *Mm*_Sp8 [GenBank: NP_796056], *Mm*_Sp9 [GenBank: NP_001005343], *Dr*_Sp1 [GenBank: NP_997827], *Dr*_Sp2 [GenBank: NP_001093452], *Dr*_Sp3 [GenBank: NP_001082967], *Dr*_Sp3-like [GenBank: XP_691096], *Dr*_Sp4 [GenBank: NP_956418], *Dr*_Sp5 [GenBank: NP_851304], *Dr*_Sp5-like [GenBank: NP_919352], *Dr*_Similar_to_Sp5 [GenBank: XP_001335730], *Dr*_Sp6 [GenBank: NP_991195], *Dr*_Sp7 [GenBank: NP_998028], *Dr*_Sp8 [GenBank: NP_998406], *Dr*_Sp8-like [GenBank: NP_991113], *Dr*_Sp9 [GenBank: NP_998125], *Gg*_Sp1 [GenBank: NP_989935], *Gg*_Sp2 [GenBank: XP_423405], *Gg*_Sp3 [GenBank: NP_989934], *Gg*_Sp4 [GenBank: XP_418708], *Gg*_Sp5 [GenBank: NP_001038149], *Gg*_Sp8 [GenBank: AAU04515.1], *Gg*_Sp9 [GenBank: AAU04516.1], Fr_Sp1 [GenBank: CAAB01000453.1], Fr_Sp2 [GenBank: CAAB01001586.1], Fr_Sp3 [GenBank: CAAB01000508.1], Fr_Sp3-like [GenBank: CAAB01000254.1], Fr_Sp4 [GenBank: CAAB01001019.1], Fr_Sp5 [GenBank: CAAB01001064.1], Fr_Sp5-like [GenBank: CAAB01000006.1], Fr_Sp6 [GenBank: CAAB01004244.1], Fr_Sp7 [GenBank: CAAB01000453.1], Fr_Sp8 [GenBank: CAAB01001019.1], Fr_Sp9 [GenBank: CAAB01000508.1]. In addition, we have provisionally annotated the Sp-family genes of *D. pulex, T. adhaerens *and *B. floridae *using the following genomic regions: *Dp_Sp1-4 *[NCBI_GNO_320154, scaffold_15:792601, 795915], *Dp_Sp5/btd *[NCBI_GNO_60744, scaffold_130:263041, 265220], *Dp_Sp6-9 *[NCBI_GNO_424374, scaffold_42:102959, 162646], *Ta_Sp1-4 *[scaffold_3:4169974, 4284735], *Ta_Sp5/btd *[scaffold_15:1197089, 1197409], *Ta_Sp6-9 *[scaffold_15:1120368, 1120718], *Bf_Sp1-4 *[Bf_V2_288:2820, 5436], *Bf_Sp5/btd *[Bf_V2_149:860371, 860057], *Bf_Sp6-9 *[Bf_V2_149:758897, 759229]. Click here for file

Additional file 2 **Genomic locations of Sp genes and Hox genes**. This table supplements the schematic overview given in Fig. 9. The first column gives the chromosome (or linkage group/scaffold) of a given species. The second column gives the Sp genes and Hox genes present on this chromosome (linkage group/scaffold); only representative Hox genes are given for reasons of clarity. The third column gives the exact location of the genes. The base pair values and genomic positions are based on the following genome assembly versions: *H. sapiens*: Genome Reference Consortium Human Build 37 (GRCh37), Primary_Assembly; *D. melanogaster*: release 5.10, *A. gambiae*: AgamP3.3, *A. mellifera*: Amel_4.0, *T. castaneum*: Tcas_3.0, *D. pulex*: JGI-2006-09, *N. vectensis*: Nematostella vectensis v1.0. The data for the *N. vectensis *Hox genes can be found in the references given in the table. Alternating shading for different species is used in the table to enhance the legibility of the table. Abbreviations: LG, linkage group; un, unassembled portions of the genome. Click here for file

Additional file 3 **Sequence information about the gene specific primers used in this study**. The first column gives the species and the gene. The second column gives the primer sequences in 5' to 3' orientation. The third column gives the length of the cloned fragment resulting from the PCR with the given primers. The fourth column gives the clone ID number. The fifth column gives the polymerase used to transcribe the RNA probe used for in situ hybridizations. The primers for *D. melanogaster *and *T. castaneum *have been designed as gene specific pairs using the genome sequence information. For *O. fasciatus*, *T. domestica*, *F. candida*, and *P. hawaiensis *we first isolated a small fragment of the genes using degenerate primers specified in the Materials and methods section. The gene specific RACE primers were designed on the basis of this sequence information and were used in conjunction with the commercial RACE adaptor primers. The cloned fragment of *Ph Sp6-9 *resulted from priming of the given primer pair. Abbreviations: Fwd, forward; Rev, reverse. Click here for file
