# Supplementary material for: A clustered set of three Sp-family genes is ancestral in the Metazoa: evidence from sequence analysis, protein domain structure, developmental expression patterns and chromosomal location
Source: BMC Evol Biol. 2010 Mar 30;10:88. doi: 10.1186/1471-2148-10-88 (PMC3087555; doi:10.1186/1471-2148-10-88)
Supplement: Additional file 1 — Sequence alignment used as basis for the phylogenetic analysis shown in Fig. 1. CLUSTAL X (1.81) multiple sequence alignment of different Sp factors comprising the conserved region of the Btd box (in blue) and the three zinc fingers (in red). Accession numbers of used Sp proteins: Dm_CG5669 [GenBank: NP_651232], Dm_Btd [GenBank: NP_511100], Dm_D-Sp1 [GenBank: NP_572579], Dps_GA19045 [GenBank: XP_001358829], Dps_GA22354 [GenBank: XP_002134535], Dps_GA12282 [GenBank: XP_001354397], Ag_Sp1-4 [GenBank: NZ_AAAB02008898], Ag_Sp5/Btd [GenBank: NZ_AAAB02008847], Ag Sp6-9 [GenBank: NZ_AAAB01008847]; Nav_Sp1-4 [GenBank: XP_001599101], Nav_Sp5/Btd [GenBank: AAZX01008599], Nav_Sp6-9 [GenBank: XP_001606079], Am_Sp1-4 [GenBank: XP_624316.2], Am_Sp5/Btd [GenBank: XP_001119912], Am_Sp6-9 [GenBank: XP_624528], Bm_Sp1-4 [GenBank: BABH01010251], Bm_Sp5/Btd [GenBank: BABH01024462], Bm_Sp6-9 [GenBank: AADK01002198], Tc_Sp1-4 [GenBank: XP_972252], Tc_Btd [GenBank: NP_001107792], Tc_Sp8 [GenBank: NP_001034509], Of_Sp8/9 [EMBL: FN396612], Nv_Sp1-4 [GenBank: XP_001635004], Nv_Sp5/Btd [GenBank: XP_001635002], Nv_Sp6-9 [GenBank: XP_001634948], Sp_Sp1-4 [GenBank: XR_025838], Sp_Sp5/Btd [GenBank: XP_789110.1], Sp_Sp6-9 [GenBank: XP_793203.2], Hs_Sp1 [GenBank: NP_612482], Hs_Sp2 [GenBank: NP_003101], Hs_Sp3 [GenBank: NP_003102], Hs_Sp4 [GenBank: NP_003103], Hs_Sp5 [GenBank: NP_001003845], Hs_Sp6 [GenBank: NP_954871], Hs_Sp7 [GenBank: NP_690599], Hs_Sp8 [GenBank: NP_874359], Hs_Sp9 [GenBank: NP_001138722], Mm_Sp1 [GenBank: NP_038700], Mm_Sp2 [GenBank: NP_084496], Mm_Sp3 [GenBank: NP_035580], Mm_Sp4 [GenBank: NP_033265], Mm_Sp5 [GenBank: NP_071880], Mm_Sp6 [GenBank: NP_112460], Mm_Sp7 [GenBank: NP_569725], Mm_Sp8 [GenBank: NP_796056], Mm_Sp9 [GenBank: NP_001005343], Dr_Sp1 [GenBank: NP_997827], Dr_Sp2 [GenBank: NP_001093452], Dr_Sp3 [GenBank: NP_001082967], Dr_Sp3-like [GenBank: XP_691096], Dr_Sp4 [GenBank: NP_956418], Dr_Sp5 [GenBank: NP_851304], Dr_Sp5-like [GenBank: NP_919352], Dr_Similar_to_Sp [file 1471-2148-10-88-S1.PDF]

Additional file 1

Btd-box (positions 1–10), ZF 1, ZF 2, ZF 3 (alignment positions 1–130)

```
                1                                                                                                                              130
Dm_D_Sp1        GRATCDCPNCQEAERLGP-------------------AGVHLRKKNIHSCHIPGCGKVYGKTSHLKAHLRWHTGERPFVCNWLFCGKRFTRSDELQRHLRTHTGEKRFACPVCNKRFMRSDHLAKHVKTH
Dps_GA11282     GRATCDCPNCQEAERLGP-------------------AGVHLRKKNIHSCHIPGCGKVYGKTSHLKAHLRWHTGERPFVCNWLFCGKRFTRSDELQRHLRTHTGEKRFACPVCNKRFMRSDHLAKHVKTH
Am_Sp6-9        GRATCDCPNCQEAERLGP-------------------AGVHLRKKNIHSCHIPGCGKVYGKTSHLKAHLRWHTGERPFVCNWLFCGKRFTRSDELQRHLRTHTGEKRFACPVCNKRFMRSDHLAKHVKTH
Nav_Sp6-9       GRATCDCPNCQEAERLGP-------------------AGVHLRKKNIHSCHIPGCGKVYGKTSHLKAHLRWHTGERPFVCNWLFCGKRFTRSDELQRHLRTHTGEKRFACPVCNKRFMRSDHLAKHVKTH
Ag_Sp6-9        GRATCDCPNCQEAERLGP-------------------AGVHLRKKNIHSCHIPGCGKVYGKTSHLKAHLRWHTGERPFVCNWLFCGKRFTRSDELQRHLRTHTGEKRFACPVCNKRFMRSDHLAKHVKTH
Td_Sp6-9        GRATCDCPNCQEAERLGP-------------------AGVHLRKKNIHSCHIPGCGKVYGKTSHLKAHLRWHTGERPFVCNWLFCGKRFTRSDELQRHLRTHTGEKRFACPVCNKRFMRSDHLAKHVKTH
Tc_Sp8          GRATCDCPNCQEAERLGP-------------------AGVHLRKKNIHSCHIPGCGKVYGKTSHLKAHLRWHTGERPFVCNWLFCGKRFTRSDELQRHLRTHTGEKRFACPICNKRFMRSDHLAKHVKTH
Of_Sp8/9        GRATCDCPNCQEAERLGP-------------------AGVHLRKKNIHSCHIPGCGKVYGKTSHLKAHLRWHTGERPFVCNWLFCGKRFTRSDELQRHLRTHTGEKRFACPVC-----------------
Bm_Sp6-9        GRATCDCPNCQEAERLGP-------------------AGAHLRKKNIHSCHIPGCGKVYGKTSHLKAHLRWHTGERPFVCNWLFCGKRFTRSDELQRHLRTHTGEKRFACPVCNKRFMRSDHLAKHVKTH
Fc_Sp6-9        GRATCDCPNCQEAERLGP-------------------AGAHLRKKNIHSCHIPGCGKVYGKTSHLKAHLRWHTGERPFVCNWLFCGKRFTRSDELQRHLRTHTGEKRFACPICNKRFMRSDHLSKHVKTH
Ph_SP6-9        GRATCDCPNCQEADRLGP-------------------AGAHLRKRNIHSCHIPGCGKVYGKTSHLKAHLRWHTGERPFVCNWLFCGKRFTRSDELQRHLRTHTGEKRFACPVCNKRFMRSDHLSKHVKTH
Dp_Sp6-9        GRATCDCPNCQEADRLGP-------------------AGAHLRKRNIHSCHIPGCGKVYGKTSHLKAHLRWHTGERPFVCNWLFCGKRFTRSDELQRHLRTHTGEKRFACPVCNKRFMRSDHLSKHVKTH
Gg_Sp9          GRATCDCPNCQEAERLGP-------------------AGASLRRKGLHSCHIPGCGKVYGKTSHLKAHLRWHTGERPFVCNWLFCGKRFTRSDELQRHLRTHTGEKRFACPVCNKRFMRSDHLSKHIKTH
Fr_Sp9          GRATCDCPNCQEAERLGP-------------------AGASLRRKGLHSCHIPGCGKVYGKTSHLKAHLRWHTGERPFVCNWLFCGKRFTRSDELQRHLRTHTGEKRFACPVCNKRFMRSDHLSKHIKTH
Dr_Sp9          GRATCDCPNCQEAERLGP-------------------AGASLRRKGLHSCHIPGCGKVYGKTSHLKAHLRWHTGERPFVCNWLFCGKRFTRSDELQRHLRTHTGEKRFACPVCNKRFMRSDHLSKHIKTH
Hs_Sp9          GRATCDCPNCQEAERLGP-------------------AGASLRRKGLHSCHIPGCGKVYGKTSHLKAHLRWHTGERPFVCNWLFCGKRFTRSDELQRHLRTHTGEKRFACPVCNKRFMRSDHLSKHIKTH
Mm_Sp9          GRATCDCPNCQEAERLGP-------------------AGASLRRKGLHSCHIPGCGKVYGKTSHLKAHLRWHTGERPFVCNWLFCGKRFTRSDELQRHLRTHTGEKRFACPVCNKRFMRSDHLSKHIKTH
Mm_Sp8          GRATCDCPNCQEAERLGP-------------------AGASLRRKGLHSCHIPGCGKVYGKTSHLKAHLRWHTGERPFVCNWLFCGKRFTRSDELQRHLRTHTGEKRFACPVCNKRFMRSDHLSKHVKTH
Hs_Sp8          GRATCDCPNCQEAERLGP-------------------AGASLRRKGLHSCHIPGCGKVYGKTSHLKAHLRWHTGERPFVCNWLFCGKRFTRSDELQRHLRTHTGEKRFACPVCNKRFMRSDHLSKHVKTH
Dr_Sp8-like     GRATCDCPNCQEAERLGP-------------------AGASLRRKGLHSCHIPGCGKVYGKTSHLKAHLRWHTGERPFVCNWLFCGKRFTRSDELQRHLRTHTGEKRFACPVCNKRFMRSDHLSKHVKTH
Gg_Sp8          GRATCDCPNCQEAERLGP-------------------AGASLRRKGLHSCHIPGCGKVYGKTSHLKAHLRWHTGERPFVCNWLFCGKRFTRSDELQRHLRTHTGEKRFACPVCNKRFMRSDHLSKHVKTH
Dr_Sp8          GRATCDCPNCQEAERLGP-------------------AGASLRRKGLHSCHIPGCGKVYGKTSHLKAHLRWHTGERPFVCNWLFCGKRFTRSDELQRHLRTHTGEKRFSCPVCNKRFMRSDHLSKHVKTH
Fr_Sp8          GRATCDCPNCQEAESLGP-------------------GGASPQRRGLHSCHIPGCGKVYGKTSHLKAHLRWHTGERPFVCNWLFCGKRFTRSDELQRHLRTHTGEKRFECAICQKRFMRSDHLSKHIRTH
Bf_SpA          GRATCDCPNCQEAERLGP-------------------AGAAIRRKNQHSCHIPGCGKVYGKTSHLKAHLRWHTGERPFVCNWLFCGKRFTRSDELQRHLRTHTGEKRFACPVCNKRFMRSDHLAKHTKTH
Sp_Sp6-9        GRATCDCPNCQENERLAA-------------------AGQPLRKKNVHSCHIPGCGKIYGKTSHLKAHLRWHTGERPFVCNWLFCGKRFTRSDELQRHLRTHTGEKRFSCPTCNKRFMRSDHLSKHVKTH
Mm_Sp7          GRSTCDCPNCQELERLGA-------------------AAAGLRKKPIHSCHIPGCGKVYGKASHLKAHLRWHTGERPFVCNWLFCGKRFTRSDELERHVRTHTREKKFTCLLCSKRFTRSDHLSKHQRTH
Hs_Sp7          GRSSCDCPNCQELERLGA-------------------AAAGLRKKPIHSCHIPGCGKVYGKASHLKAHLRWHTGERPFVCNWLFCGKRFTRSDELERHVRTHTREKKFTCLLCSKRFTRSDHLSKHQRTH
Dr_Sp7          GRSSCDCPNCQELERLGA-------------------SAASLRKKPVHSCHIPGCGKVYGKASHLKAHLRWHTGERPFVCNWLFCGKRFTRSDELERHVRTHTREKKFTCLLCNKRFTRSDHLSKHQKTH
Fr_Sp7          TRSSCDCPNCQELERLGA-------------------SAASLRKKPVHSCHIPGCGKVYGKASHLKAHLRWHTGERPFVCNWLFCGKRFTRSDELERHVRTHTREKKFTCLLCNKRFTRSDHLSKHQKTH
Nv_Sp6-9        GRATCDCPNCQDNERMSA-------------------SGAPFRKKSQHICHIPGCGKVYGKTSHLKAHLRWHTGERPFVCNWLFCGKRFTRSDELQRHLRTHTGEKRFACPICSKRFMRSDHLSKHVKTH
Mm_Sp6          GQTVCRCPNCLEAERLGAP-----------------CGPDGGKKKHLHNCHIPGCGKAYAKTSHLKAHLRWHSGDRPFVCNWLFCGKRFTRSDELQRHLQTHTGTKKFPCAVCSRVFMRSDHLAKHMKTH
Hs_Sp6          GQTVCRCPNCLEAERLGAP-----------------CGPDGGKKKHLHNCHIPGCGKAYAKTSHLKAHLRWHSGDRPFVCNWLFCGKRFTRSDELQRHLQTHTGTKKFPCAVCSRVFMRSDHLAKHMKTH
Dr_Sp6          GQASCRCPNCLSAESLGN-------------------SGDASKRKHLHNCHIPGCGKAYVKTSHLKAHLRWHSGDRPFVCNWLFCGKRFTRSDELQRHLQTHTGAKRFGCSSCPRVFLRADHLAKHMRVH
Fr_Sp6          AQAVCRCPNCIHAEQLGQ-------------------STDDTRRKHMHNCHIPGCGKAYAKTSHLKAHLRWHSGDRPFVCNWLFCGKRFTRSDELQRHLQTHTGAKKFSCALCPRVFMRNDHLAKHMRTH
Ta_Sp6-9        NRSSCNCPYCQENERLAAT------------------GQPVPRRKAQHICHIADCGKVYTKTSHLKAHLRWHSGERPFVCNWLFCGKRFTRSDELQRHLRTHTGEKRFVCKVCSKRFMRSDHLSKHMKTH
Bm_Sp5/Btd      RCAKCRCPNCLTEAAGFGP------------------NYGKDGAKREHVCHVPGCGKVYGKTSHLKAHLRWHTGERPFVCNWLFCGKRFTRSDELQRHLRTHTGEKRFSCQLCTKRFMRSDHLAKHVKTH
Tc_Btd          RCIKCQCPNCVNEEVGLK----------------------KPSSKKVHVCHYQGCDKVYGKTSHLQAHLRWHTGERPFVCNWLFCGKRFTRSDELQRHLRTHTGEKRFACSVCTKRFMRSDHLAKHVKTH
Fc_Sp5/Btd      KSRRCRCPNCLAGIQPTQV------------------P-GEKKKKRQHICHMIGCGKIYGKTSHLKAHLRGHTGERPFVCNWLFCGKRFTRSDELQRHLRTHTGEKRFICNVCQKRFMRSDHLAKHVKTH
Am_Sp5/Btd      KCIRCRCPNCQTENGGSQL------------------G---LDGKRQHVCHVPGCGKVYGKTSHLKAHLRWHTGERPFVCNWLFCGKRFTRSDELQRHLRTHTGEKRFACPTCGKRFMRSDHLTKHVKTH
Nav_Sp5/Btd     KCTRCQCPNCMADGG-----------------------NVGRDGKRQHSCHIDGCNKVYGKTSHLKAHLRWHSGEKPYFCNWKPCGKSFTRSDELQRHMRTHTGEKRFVCPECGKRFMRSDHLNKHRKTH
Dr_Sim-to-Sp5   QSRRCMCPNCQKNAD-----------------------TPGR--RKQHACHIPGCAKVYGKTSHLKAHLRWHAGERPFICSWMFCGKSFTRSDELQRHLRTHTGEKRFTCPDCSKRFMRSDHLAKHLKTH
Dr_Sp5          RCRRCRCPNCQSSS-----------------------SSDEPGKKKQHICHIPGCGKVYGKTSHLKAHLRWHSGERPFVCNWLFCGKSFTRSDELQRHLRTHTGEKRFVCPDCCKRFMRSDHLAKHVKTH
Fr_Sp5          RCRRCRCPNCQSST-----------------------SSDEPGKKKQHICHIPGCGKVYGKTSHLKAHLRWHSGERPFVCNWLFCGKSFTRSDELQRHLRTHTGEKRFVCPDCCKRFMRSDHLAKHVKTH
Bf_Sp5          RCRRCRCPNCQNST-----------------------AGS-PNKKKQHICHIPGCGKVYGKTSHLKAHLRWHTGERPFVCNWLFCGKSFTRSDELQRHLRTHTGEKRFACPDCGKRFMRSDHLAKHVKTH
Sp_Sp5/Btd      RCRRCRCPNCLNPS-----------------------ISSEPAKKRQHICHIPGCGKVYGKTSHLKAHLRWHTGERPFVCNWLFCGKSFTRSDELQRHLRTHTGEKRFVCPECGKRFMRSDHLSKHSKTH
Dp_Sp5/Btd      RCRRCRCPNCQDPTTGASIT----------------GHSNSQHKKKQHLCHVPGCGKVYGKTSHLKAHLRWHAGERPFSCQWLFCGKSFTRSDELQRHLRTHTGEKRFACPVCNKRFMRSDHLSKHSKTH
Mm_Sp5          RCRRCRCPNCQAAGG---------------------APEAEPGKKKQHVCHVPGCGKVYGKTSHLKAHLRWHTGERPFVCNWLFCGKSFTRSDELQRHLRTHTGEKRFACPECGKRFMRSDHLAKHVKTH
```

```
Hs_Sp5        RCRRCRCPNCQAAGG---------------------APEAEPGKKKQHVCHVPGCGKVYGKTSHLKAHLRWHTGERPFVCNWLFCGKSFTRSDELQRHLRTHTGEKRFACPECGKRFMRSDHLAKHVKTH
Gg_Sp5        RCRRCRCPNCQAAAGS--------------------APEAEPGKKKQHVCHIPGCGKVYGKTSHLKAHLRWHTGERPFVCNWLFCGKSFTRSDELQRHLRTHTGEKRFVCPECGKRFMRSDHLAKHVKTH
Nv_Sp5/Btd    RCRRCRCPNCQLAAT-----------------------TGNTSKRKQHVCHIPGCGKVYGKTSHLKAHLRWHTGERPFVCNWLFCGKSFTRSDELQRHLRTHTGEKRFQCDECGKRFMRSDHLRKHQKTH
Dr_Sp5-like   RCRRCKCPNCQSTGNGG--------------------AALEFGKKRLHICHIPDCGKVYKKTSHLKAHLRWHAGERPFICNWLFCGKSFTRSDELQRHLRTHTGEKRFGCQQCGKRFMRSDHLSKHVKTH
Fr_Sp5-like   RCRRCKCPNCQANGGG-----------------------LEFGKKRLHVCHIPDCGKVYKKTSHLKAHLRWHAGERPFICNWLFCGKSFTRSDELQRHLRTHTGEKRFGCQQCGKRFMRSDHLSKHVKTH
Td_Sp5/Btd    RCSRCRCPNCQNGGN---------------------TAGQQPQRKRQHVCHVPGCSKTYGKTSHLKAHLRWHTGERPFVCNWLFCGKSFTRSDELQRHLRTHTGEKRFACPVCNKRFMRSDHLAKHVKTH
Mm_Sp2        RRMACTCPNCKDGEK-----------------------RSGEQGKKKHVCHIPDCGKTFRKTSLLRAHVRLHTGERPFVCNWFFCGKRFTRSDELQRHARTHTGDKRFECAQCQKRFMRSDHLTKHYKTH
Hs_Sp2        RRMACTCPNCKDGEK-----------------------RSGEQGKKKHVCHIPDCGKTFRKTSLLRAHVRLHTGERPFVCNWFFCGKRFTRSDELQRHARTHTGDKRFECAQCQKRFMRSDHLTKHYKTH
Gg_Sp2        RRMACTCPNCKDGEK-----------------------RPGDQGKKKHICHIPECGRTFRKTSLLRAHVRLHTGERPFVCNWVFCGKRFTRSDELQRHARTHTGDKRFECAQCQKRFMRSDHLTKHYKTH
Dr_Sp2        RHISCLCFLCY---------------------------RPGEVGKRKHICHIAGCEKTFRKTSLLRAHVRLHTGERPFVCNWVFCGKRFTRSDELQRHARTHTGKKRFECNKCQKRFMRSDHLTKHYKTH
Fr_Sp2        RRMACTCPNCKDADK-----------------------RPGEVGKRKHICHVPGCEKTFRKTSLLRAHVRLHTGERPFVCSWVFCGKRFTRSDELQRHARTHTGDKRFECSQCQKRFMRSDHLTKHYKTH
Ta_Sp1-4      ----------------------------------------------NHLSHL----------SLLKAHQQAKVGERPFPCTWSECGKRFARSDELARHYRTHTGEKRFECPVCNKRFMRSDHLSKHAKRH
Dm_CG5669     KRVACTCPNCTDGEKHS-------------------------DKKRQHICHITGCHKVYGKTSHLRAHLRWHTGERPFVCSWAFCGKRFTRSDELQRHRRTHTGEKRFQCQECNKKFMRSDHLSKHIKTH
Dps_GA19045   KRVACTCPNCTDGEKHS-------------------------DKKRQHICHIPGCQKVYGKTSHLRAHLRWHTGERPFVCSWVFCGKRFTRSDELQRHRRTHTGEKRFQCRECNKKFMRSDHLSKHIKTH
Fc_Sp1-4      RRVACTCPNCTDIDASSNNSRN------------------NGEKKKQHLCHIPGCGKVYGKTSHLRAHLRWHAGERPFVCQWLFCGKRFTRSDELQRHRKTHTGEKRFQCPECLKKFMRSDHLSKHIKTH
Am_Sp1-4      RRVACTCPNCGDGDRNRD-----------------------MTRKRQHVCHIAGCNKVYGKTSHLRAHLRWHTGERPFVCSWIFCGKKFTRSDELQRHRRTHTGEKRFQCPECTKKFMRSDHLTKHIKTH
Nav_Sp1-4     RRVACTCPNCGDGDRNRD-----------------------MTRKRQHVCHIPGCSKVYGKTSHLRAHLRWHTGERPFVCNWIFCGKKFTRSDELQRHRRTHTGEKRFECPECTKKFMRSDHLTKHIKTH
Tc_Sp1-4      RRVACTCPNCQEGERHAD-------------------------RKKQHICHIPGCNKVYGKTSHLRAHLRWHTGERPFVCSWLFCGKRFTRSDELQRHRRTHTGEKRFQCNECSKKFMRSDHLSKHLKTH
Ag_Sp1-4      RRVACTCPNCEVKSSGPP------------------------DRKRQHICHVSGCNKVYGKTSHLRAHLRWHTGERPFVCSWLFCGKRFTRSDELQRHRRTHTGEKRFQCNECSKKFMRSDHLSKHIRTH
Of_Sp1-4      RRVACSCPNCIEGHAGCSRLRAEEATTSLEHVRTINKATWGGIRKKQHVCHVAGCNKVYGKTSHLRAHLRWHTGERPFICNWGTCGKRFTRSDELQRHRRTHTGEKRFECVECNKKFMRSDHLSKHIRTH
Bm_Sp1-4      KRVACTCPNCDQGENVFSR---------------------VVDRKKQHVCHIAGCNKVYGKTSHLRAHLRWHTGERPFICSWEFCGKCFTRSDELQRHRRTHTGEKRFMCTQCHKKFMRSDHLAKHVRIH
Td_Sp1-4      -------------------------------------------------------NKVYGKTSHLRAHLRWHSGERPFLCNWLFCGKRFTRSDELQRHRRTHTGEKRFECPECSKRFMRSDHLSKHIKTH
Mm_Sp1        RREACTCPYCKDSEGRASG---------------------DPGKKKQHICHIQGCGKVYGKTSHLRAHLRWHTGERPFVCTWMFCGKRFTRSDELQRHRRTHTGEKRFQCPECSKKFMRSDHLSKHIKTH
Hs_Sp1        RREACTCPYCKDSEGRGSG---------------------DPGKKKQHICHIQGCGKVYGKTSHLRAHLRWHTGERPFMCNWSYCGKRFTRSDELQRHKRTHTGEKKFACPECPKRFMRSDHLSKHIKTH
Gg_Sp1        RREACTCPYCKDSEGRSSG---------------------DPGKKKQHICHIPGCGKVYGKTSHLRAHLRWHTGERPFICGWMLCGKRFTRSDELQRHKRTHTGEKKFACPECPKRFMRSDHLSKHIKTH
Dr_Sp1        RREACTCPYCKDGEGR------------------------DPSKKKQHICHIPGCGKVYGKTSHLRAHLRWHTGERPFVCSWSFCGKRFTRSDELQRHKRTHTGEKKFSCTECPKRFMRSDHLSKHIKTH
Fr_Sp1        RREACTCPFCKDGEGR------------------------DPTKKKQHICHISGCGKIYGKTSHLRAHLRWHTGERPFVCSWSFCGKRFTRSDELQRHKRTHTGEKRFACPECPKRFMRSDHLSKHIKTH
Dp_Sp1-4      RRVACTCPNCKDGEVARKSNRPK-----------IFPSRVGENKRKIHICHHQGCGKVYGKTSHLRAHLRWHSGDRPFVCSWYYCGKRFTRSDELQRHRRTHTGEKRFQCPECGKRFMRSDHLSKHIKTH
Mm_Sp3        RRVACTCPNCKEGGGRGTN----------------------LGKKKQHICHIPGCGKVYGKTSHLRAHLRWHSGERPFICNWMFCGKRFTRSDELQRHRRTHTGEKKFVCPECSKRFMRSDHLAKHIKTH
Hs_Sp3        RRVACTCPNCKEGGGRGTN----------------------LGKKKQHICHIPGCGKVYGKTSHLRAHLRWHSGERPFVCNWMYCGKRFTRSDELQRHRRTHTGEKKFVCPECSKRFMRSDHLAKHIKTH
Gg_Sp3        RRVACTCPNCKEGGGRGSN----------------------LGKKKQHICHIPGCGKVYGKTSHLRAHLRWHSGERPFVCNWMFCGKRFTRSDELQRHRRTHTGEKKFVCPECSKRFMRSDHLAKHIKTH
Dr_Sp3        RRVACTCPNC-EAGGRGSS----------------------MGKKKQHICHIPGCGKVYGKTSHLRAHLRWHSGERPFICSWSYCGKRFTRSDELQRHRRTH-GEKKFVCPECSKRFMRSDHLAKHIKTH
Dr_Sp3-like   RRVACTCPNCKEAGGRGSS----------------------MGKKKQHICHIPGCGKVYGKTSHLRAHLRWHSGERPFICSWSYCGKRFTRSDELQRHRRTHTGEKKFVCPECSKRFMRSDHLAKHIKTH
Fr_Sp3        RRVACTCPNCKESGGRGSS----------------------TGKKKQHICHIAGCGKVYGKTSHLRAHLRWHSGERPFVCSWMLCGKRFTRSDELQRHRRTHTGEKKFVCPECSKRFMRSDHLAKHIKTH
Fr_Sp3-like   RRVACTCPNCKESGGRGSG----------------------MGKKKQHICHIAGCGKVYGKTSHLRAHLRWHSGERPFVCNWMFCGKRFTRSDELQRHRRTHTGEKKFVCMECSKRFMRSDHLAKHIKTH
Sp_Sp4        RRLACTCPNCKDGDGRG---------------------------KKQHICHIADCGKIYGKTSHLRAHLRWHTGERPFVCDWLFCGKRFTRSDELQRHRRTHTGEKKFVCKSCGKKFMRSDHLAKHQRTH
Mm_Sp4        RRVACSCPNCREGEGRGSS---------------------EPGKKKQHVCHIEGCGKVYGKTSHLRAHLRWHTGERPFICNWMFCGKRFTRSDELQRHRRTHTGEKRFECPECSKRFMRSDHLSKHVKTH
Hs_Sp4        RRVACSCPNCREGEGRGSN---------------------EPGKKKQHICHIEGCGKVYGKTSHLRAHLRWHTGERPFICNWMFCGKRFTRSDELQRHRRTHTGEKRFECPECSKRFMRSDHLSKHVKTH
Gg_Sp4        RRVACSCPNCREGEGRSSN---------------------EPGKKKQHICHIEGCGKVYGKTSHLRAHLRWHTGERPFICNWVFCGKRFTRSDELQRHRRTHTGEKRFECPECSKRFMRSDHLSKHVKTH
Dr_Sp4        RRVACSCPNCRDGEGRNNS---------------------DPSKKKQHVCHMEGCGKVYGKTSHLRAHLRWHTGERPFVCNWIFCGKRFTRSDELQRHRRTHTGEKRFECPECSKRFMRSDHLSKHIKTH
Fr_Sp4        RRVACSCPNCRDGEGRNSG---------------------DPTKKKQHICHMEGCGKVYGKTSHLRAHLRWHTGERPFVCNWIFCGKRFTRSDELQRHRRTHTGEKRFECPECSKRFMRSDHLSKHIKTH
Bf_SpB        RRVACSCPNCREGEGRGNG---------------------D-SKKKQHICHIAGCGKVYGKTSHLRAHLRWHTGERPFVCNWLFCGKRFTRSDELQRHRRTHTGEKRFTCPDCSKKFMRSDHLSKHIKTH
Ph_Sp1-4      KRVACTCPNCREGGVNNERGEPSSSNGI-----------GGSTKRRQHICHIPGCNKTYGKTSHLRAHLRWHAGDRPFACNWMFCTKRFTRSDELQRHKRTHTGEKRFHCPDCDKKFMRSDHLSKHVKTH
Nv_Sp1-4      -RIACTCPNCRDG-------EGRTANG-----------------RKQHVCHVPGCGKVYGKTSHLRAHLRWHSGERPFVCNWLFCGKRFTRSDELQRHRRTHTGEKRFTCPECNKKFMRSDHLSKHVKTH
Ta_Sp5/Btd    RRVACCCPNCRNPDYKPPQ-----------------------GGKKMHVCHYQGCSKVYGKTSHLRAHLRWHTGERPFVCNWLFCGKRFTRSDELQRHRRTHTGEKKFACQDCGKRFMRSDHLSKHVKTH
Dm_Btd        RSVRCTCPNCTNEMSGLPP-----------------IVGPDERGRKQHICHIPGCERLYGKASHLKTHLRWHTGERPFLC--LTCGKRFSRSDELQRHGRTHTNYRPYACPICSKKFSRSDHLSKHKKTH
Dps_GA11738   RSVRCTCPNCTNEMSGLPP-----------------IVGPDERGRKQHICHIPGCERLYGKASHLKTHLRWHTGERPFLC--LTCGKRFSRSDELQRHGRTHTNYRPYACPICSKKFSRSDHLSKHKKTH
Ag_Sp5/Btd    RCARCTCPNCINELSGLPP-----------------VVGPDEKGKRQHICHIPGCEKIYGKTSHLKAHLRWHTGERPFQCKWLFCGKRFTRSDELQRHFRTHTGEKRFTCTICSKKFMRSDHLAKHVKTH
```
