# Supplementary material for: A clustered set of three Sp-family genes is ancestral in the Metazoa: evidence from sequence analysis, protein domain structure, developmental expression patterns and chromosomal location
Source: BMC Evol Biol. 2010 Mar 30;10:88. doi: 10.1186/1471-2148-10-88 (PMC3087555; doi:10.1186/1471-2148-10-88)
Supplement: Additional file 2 — Genomic locations of Sp genes and Hox genes. This table supplements the schematic overview given in Fig. 9. The first column gives the chromosome (or linkage group/scaffold) of a given species. The second column gives the Sp genes and Hox genes present on this chromosome (linkage group/scaffold); only representative Hox genes are given for reasons of clarity. The third column gives the exact location of the genes. The base pair values and genomic positions are based on the following genome assembly versions: H. sapiens: Genome Reference Consortium Human Build 37 (GRCh37), Primary_Assembly; D. melanogaster: release 5.10, A. gambiae: AgamP3.3, A. mellifera: Amel_4.0, T. castaneum: Tcas_3.0, D. pulex: JGI-2006-09, N. vectensis: Nematostella vectensis v1.0. The data for the N. vectensis Hox genes can be found in the references given in the table. Alternating shading for different species is used in the table to enhance the legibility of the table. Abbreviations: LG, linkage group; un, unassembled portions of the genome. [file 1471-2148-10-88-S2.PDF]

**species/location**	**Sp genes/Hox**	**Chromosomal position**
Human<br>Chromosome 2q	*Sp3*<br>*Sp5*<br>*Sp9*<br>HOXD1<br>HOXD13	174.773-174.830 kbp<br>171.571-171.574 kbp<br>175.199-175.202 kbp<br>177.053-177.055 kbp<br>176.957-176.960 kbp
Human<br>Chromosome 7p	*Sp4*<br>*Sp8*<br>HOXA1<br>HOXA13	21.467-21.554 kbp<br>20.821-20.826 kbp<br>27.132-17.135 kbp<br>27.236-27.239 kbp
Human<br>Chromosome 12q	*Sp1*<br>*Sp7*<br>HOXC4<br>HOXC13	53.773-53.810 kbp<br>53.720-53.729 kbp<br>54.410-54.449 kbp<br>54.332-54.304 kbp
Human<br>Chromosome 17q	*Sp2*<br>*Sp6*<br>HOXB1<br>HOXB13	45.973-46.006 kbp<br>45.922-45.933 kbp<br>46.606-46.608 kbp<br>46.802-46.806 kbp
Drosophila<br>X-chromosome	*D-Sp1* (*Sp6-9*)<br>*btd*	9.623-9.649 kbp<br>9.588-9.591 kbp
Drosophila<br>Chromosome 3R	CG5669 (*Sp1-4*)<br>Antennapedia complex<br>Bithorax complex	20.046-20.052 kbp<br>2.487-2.824 kbp<br>12.482-12.797 kbp
Anopheles<br>X-chromosome	*Sp6-9*<br>*Sp5/btd*	13.344-13.349kbp<br>13.439-13.440kbp
Anopheles<br>Chromosome 2R	*Sp1-4*<br>*scr*<br>*ubx*	57.814-57.831kbp<br>59.732-59.775kbp<br>60.110k-60.173kbp
Apis<br>LG13	*Sp6-9*<br>*Sp5/btd*	1.371-1.393kbp<br>1.441-1.442kbp
Apis LGun	*Sp1-4*	8352-11768bp
Apis LG16	HOX (*labial*)	3.892-3.909kbp
Tribolium LG9	*Sp8*<br>*btd*	19.911-19.927kbp<br>19.960-19.961kbp
Tribolium LGun	*Sp1-4*	<618-6806bp
Tribolium LG2	HOX (*labial*)<br>(*Abd-B*)	10.378-10.410kbp<br>9.691-9.700kbp
Daphnia<br>Chromosome1	*Sp6-9*<br>*Sp5/btd*	102.625-163.646bp<br>263.041-281.587bp
Daphnia<br>Chromosome5	*Sp1-4*	781.813-986.944bp
Daphnia<br>Chromosome8	HOX	~544kbp
Nematostella<br>Scaff.53	*Sp1-4*<br>*Sp5/btd*<br>*Sp6-9*	330.760-331.110bp<br>305.778-306.053<br>275.750-276.850
Nematostella<br>Scaff.3, 61, 4	HOX	[49,50]

Additional file 2
