# Supplementary material for: A clustered set of three Sp-family genes is ancestral in the Metazoa: evidence from sequence analysis, protein domain structure, developmental expression patterns and chromosomal location
Source: BMC Evol Biol. 2010 Mar 30;10:88. doi: 10.1186/1471-2148-10-88 (PMC3087555; doi:10.1186/1471-2148-10-88)
Supplement: Additional file 3 — Sequence information about the gene specific primers used in this study. The first column gives the species and the gene. The second column gives the primer sequences in 5' to 3' orientation. The third column gives the length of the cloned fragment resulting from the PCR with the given primers. The fourth column gives the clone ID number. The fifth column gives the polymerase used to transcribe the RNA probe used for in situ hybridizations. The primers for D. melanogaster and T. castaneum have been designed as gene specific pairs using the genome sequence information. For O. fasciatus, T. domestica, F. candida, and P. hawaiensis we first isolated a small fragment of the genes using degenerate primers specified in the Materials and methods section. The gene specific RACE primers were designed on the basis of this sequence information and were used in conjunction with the commercial RACE adaptor primers. The cloned fragment of Ph Sp6-9 resulted from priming of the given primer pair. Abbreviations: Fwd, forward; Rev, reverse. [file 1471-2148-10-88-S3.PDF]

Gene	Gene specific primers used	Clone length	Clone ID	RNA polymerase for antisense transcription
Dm Sp1-4 (CG5669)	Fwd CCAATCTCCTGGCAGAGTAGCGCGTCTG<br>Rev CAATTGGGACAGGTGCAGGCTACTCGC	2280bp	3099	T7
Dm btd	Fwd CGCAAGCGTTTTCAACATGATCGATGCGG<br>Rev GGAGCGGCGTTGCAATTGCGTCTGGAAGG	928bp	3091	Sp6
Dm D-Sp1	Fwd TTGGAGCGCTGTGCGCGGACAGCCATAG<br>Rev CCCGTCCGGCATAACGCCTCTGGGATC	718bp	3096	Sp6
Tc Sp1-4	Fwd GTCTGGGTAACGTGCAAGTAATCGCGC<br>Rev GCAGACTGTGTCGACGTTGCAGCTTCC	748bp	3089	Sp6
Tc btd	Fwd GTGACTACTATGATGGCTTCCTCGGTCC<br>Rev CACCTCAAGTGGGCCTGGAGATGCGAGG	509bp	3077	T7
Tc Sp8	Fwd CGCATGCTGTTCCGGTCCATCTCATCGC<br>Rev GGCCAGTGTAACGTCTCTGTGACCTGG	1064bp	3105	Sp6
Of Sp1-4	3'RACE GGCCATGCTGGTTGTTCTCGTCTTCGGGCTG	594bp	3011	Sp6
Of Sp8/9	5'RACE CAGGTGAGCCTTGAGGTGCGAGGTC	1078bp	2287	Sp6
Td Sp1-4	3'RACE CACGGGAGAACGACCTTTTGTCTGTACCTGG	647bp	4121	Sp6
Td Sp5/btd	3'RACE CCCTGGGTGCTCAAAGACGTACGG	774bp	2464	Sp6
Td Sp6-9	3'RACE CCAGGTTGTGGTAAGGTATACGGG	772bp	2427	Sp6
Fc Sp1-4	3'RACE CAGAGGCATAGAAGGACGCACACGGGAGAG	737bp	1513	T7
Fc Sp5/btd	3'RACE GCAATGTATGCCAAAAACGGTTCATGCGCACC	1369bp	1503	T7
Fc Sp6-9	3'RACE GGCCCAGCAGGAGCTCACCTTAGG	736bp	1541	T7
Ph Sp1-4	3'RACE ATGGCATGCTGGTGACAGACCTTTTGCG	1799bp	117	T7
Ph Sp6-9	Fwd CGAGAAGAACGAACGGATCC<br>Rev CATGTTGGTGCTCAGTGAC	1502bp	412	Sp6

Additional file 3
